# Covariate adjustment in Bayesian adaptive randomized controlled trials

**DOI:** 10.1177/09622802241227957

**Published:** 2024-02-07

**Authors:** James Willard, Shirin Golchi, Erica EM Moodie

**Affiliations:** Epidemiology and Biostatistics, 5620 McGill University, Montreal, Canada

**Keywords:** Bayesian adaptive designs, covariate adjustment, clinical trials, power, stopping criteria

## Abstract

In conventional randomized controlled trials, adjustment for baseline values of covariates known to be at least moderately associated with the outcome increases the power of the trial. Recent work has shown a particular benefit for more flexible frequentist designs, such as information adaptive and adaptive multi-arm designs. However, covariate adjustment has not been characterized within the more flexible Bayesian adaptive designs, despite their growing popularity. We focus on a subclass of these which allow for early stopping at an interim analysis given evidence of treatment superiority. We consider both collapsible and non-collapsible estimands and show how to obtain posterior samples of marginal estimands from adjusted analyses. We describe several estimands for three common outcome types. We perform a simulation study to assess the impact of covariate adjustment using a variety of adjustment models in several different scenarios. This is followed by a real-world application of the compared approaches to a COVID-19 trial with a binary endpoint. For all scenarios, it is shown that covariate adjustment increases power and the probability of stopping the trials early, and decreases the expected sample sizes as compared to unadjusted analyses.

## 1. Introduction

In conventional, fixed-size randomized controlled trials (RCTs), adjustment for baseline values of covariates known to be at least moderately associated with the outcome has been shown to increase the power of the trial.^1–4^ This is because covariate adjustment improves the precision of the estimated treatment effect and accounts for the outcome heterogeneity within each treatment arm that is explained by the adjustment variables.^3,5,6^ Adjustment also corrects for any chance imbalance of important baseline variables which may exist post-randomization.^7^ Therefore, covariate adjustment in the primary analysis of clinical trials is now recommended by both the US Food and Drug Administration (FDA) and European Medicines Agency.^8,9^ Additionally, systematic reviews have suggested its use in practice has grown over time.^10,11^ Recently, these power increases have been demonstrated in more flexible frequentist designs, such as information adaptive and adaptive multi-arm designs.^12,13^ However, the simulation scenarios investigated in each of these designs contained at least one of the following: only continuous outcomes with no treatment–covariate interactions where the marginal and conditional estimands are the same; only a single sample size; a data-generating process containing only a small number of variables; or only a small number of covariate adjustment models. A more comprehensive investigation is needed to better understand the benefits of covariate adjustment under a broader array of flexible design scenarios.

While the impact of covariate adjustment has been demonstrated in the flexible frequentist designs mentioned above, it has not been characterized within flexible Bayesian adaptive designs, where early stopping at an interim analysis is permitted given evidence of treatment superiority or futility. In these designs, one may learn about a treatment effect while potentially requiring fewer participants than fixed designs. Additionally, Bayesian trials allow for seamless incorporation of prior information for model parameters including covariate effects. The impact of combining prior information with covariate adjustment has not been previously investigated. With the growing interest in Bayesian adaptive designs, a characterization of covariate adjustment for several commonly used sample sizes and outcome types would be highly valuable for researchers.

In this work, we consider the impact of covariate adjustment in Bayesian adaptive designs, which allow for early stopping for superiority, and provide a step-by-step tutorial for post-adjustment marginalization. We explore several data-generating processes for continuous, binary, and time-to-event outcomes, and consider adjustment models which include several forms of misspecification while incorporating varying levels of prior information for the covariate effects. The covariate adjustment described herein is performed using generalized linear models (GLMs). However, the methods, results, and recommendations discussed below are not specific to these models and are expected to generalize to other parametric and nonparametric models.

The article is organized in the following manner. We first introduce and describe Bayesian adaptive designs which allow for early stopping, as well as the targets of inference (i.e. estimands), which are marginal treatment effects for each endpoint. We then describe the specific collapsible and non-collapsible estimands used in this article. For the non-collapsible estimands, we describe their estimation and marginalization through a Bayesian framework. This is followed by a simulation study, which shows the impact of covariate adjustment on design operating characteristics including power, the probability of stopping the trial early, and expected sample size, for multiple sample sizes. We then show a real-world application of covariate adjustment within a COVID-19 RCT and end with a discussion.

## 2. Bayesian adaptive designs with early stopping

Bayesian adaptive designs allow for predetermined changes to the trial design at interim analyses based on evidence provided by the accumulating data.^14,15^ These designs include sequentially randomized trials, which allow for early stopping for superiority or futility at interim analyses. Interim analyses are performed to determine whether to stop the trial early and declare treatment superiority or futility or to continue the trial. The decision to stop the trial early at an interim analysis is controlled by a predefined decision rule. Decision criteria may be defined with respect to different functionals of the posterior distribution of the parameter of interest or estimand. Posterior or posterior predictive probability statements about the estimand are commonly used statistics. In the RCT setting, estimands are typically defined as marginal treatment effects in a specified population of interest. We adopt this convention throughout, but delay further discussion of marginal estimands and their posterior estimation until the next section.

In the Bayesian adaptive designs described in this work, interim or final decisions are defined with respect to posterior probability statements about a marginal treatment effect, γ(θ) , which is a function of model parameters θ . The alternative hypothesis of the trial is formulated as this marginal treatment effect being greater than a clinically meaningful threshold, $\gamma_{0}$ :

$$H_{0}:\gamma (\theta )\leq \gamma_{0}\;\text{versus}\;H_{A}:\gamma (\theta )>\gamma_{0}$$

A Bayesian test statistic can be defined as the posterior probability of this alternative hypothesis given the data, $D_{n_{t}}=\{Y_{n_{t}},A_{n_{t}},X_{n_{t}\times p}\}$ , which may include any observed outcomes ( $Y_{n_{t}}$ ), treatment assignments ( $A_{n_{t}}$ ), and p additional covariates ( $X_{n_{t}\times p}$ ), for the $n_{t}$ participants who are enrolled in the trial at an interim or final analysis conducted at time t :

(1) $$T(D_{n_{t}})=P(H_{A}|D_{n_{t}})=P(\gamma (\theta )>\gamma_{0}|D_{n_{t}})$$

This statistic is then used to define a decision rule, which declares treatment superiority at any interim or final analysis if the statistic exceeds some upper probability threshold, u , that is, if

$$T(D_{n_{t}})>u$$

If a superiority declaration is made at an interim analysis, the trial stops early. The common approach in the design of Bayesian adaptive trials is the “hybrid” approach, where the upper probability threshold, u , is optimized so that the trial design has desirable values of frequentist operating characteristics.^14^ For example, power ( P ) and the Type 1 error rate (T1E) are defined as follows:

$$\begin{array}{rl} P & =P(T(D_{n_{t}})>u|\gamma (\theta )=\gamma^{*}>\gamma_{0})\\ \text{T1E} & =P(T(D_{n_{t}})>u|\gamma (\theta )=\gamma_{0}) \end{array}$$

Since the sampling distribution of a Bayesian posterior probability is generally unknown, calibration of the design to meet frequentist operating characteristics requires simulation studies. Note that models that adjust for covariates under most settings result in analytically intractable posterior distributions. Therefore, the evaluation of $T(D_{n_{t}})$ within every trial simulation requires posterior sampling or approximation techniques. In this article, we use Markov chain Monte Carlo to sample from the posterior distribution when not available in closed form.

## 3. Estimands and Bayesian estimation

In what follows, let $A_{i}$ be defined as a binary treatment assignment for the ith participant, where $A_{i}=1$ represents being randomized to the treatment group and $A_{i}=0$ to the control group. Let ${\tilde{X}}_{n_{t}\times d}$ represent a matrix of j=1,…,d covariates measured at baseline for $i=1,\ldots ,n_{t}$ participants in the study at an interim or final analysis conducted at time t . Let ${\tilde{x}}_{i}=(x_{1},x_{2},\ldots ,x_{d}{)}_{i}$ represent the row of this matrix corresponding to the full covariate pattern of the ith participant, and let $Y_{i}$ be an arbitrary outcome of interest for the ith participant. For notational simplicity, subscripts are dropped for the remainder of this article except when strictly necessary. Treatments are assigned through simple randomization and are thus independent of all covariates measured at baseline (i.e. $A⫫\tilde{x})$ , but we allow for the possibility of chance imbalance of covariates between treatment groups.

We use the term “unadjusted analysis” to refer to a model which includes only the treatment assignment indicator ( A ), and the term “adjusted analysis” for a model which includes p≤d additional covariates, $X\subseteq \tilde{X}$ , with $x_{i}=(x_{1},\ldots ,x_{p}{)}_{i}$ representing the row of X corresponding to the ith participant’s covariate pattern used for adjustment. For adjusted analyses, many different covariate sets may be adjusted for in addition to the binary treatment indicator. This includes any covariates Z⊆X , where a treatment–covariate interaction exists. Let ϕ be the regression coefficient for the treatment assignment indicator, $\beta_{0}$ be the intercept, $\beta =\{\beta_{1},\ldots ,\beta_{p}\}$ be the vector of covariate main effects, and $\omega =\{\omega_{1},\ldots ,\omega_{m}\}$ be the vector of treatment–covariate interaction effects for those covariates used in the adjustment model within a GLM setting. Additionally, let ζ be a set of nuisance parameters not of direct interest but which are required for model specification (e.g. baseline hazard parameters in a time-to-event setting). Then let $\theta =\{\beta_{0},\phi ,\beta ,\omega ,\zeta \}$ be the set of model parameters with prior p(θ) , and define $\eta (\theta ;A_{i},X_{i},Z_{i})$ to be the expected outcome for participant i on the linear scale. Let $p(Y_{i}|A_{i},X_{i},\theta )$ represent each participant’s contribution to the likelihood function and g(⋅) be the link function. Assuming independence among participants, we have the joint posterior distribution of the model parameters, $\pi (\theta |D_{n_{t}})$ , under arbitrary adjustment model specification, being proportional to the likelihood times the prior:

$$\begin{array}{rl} \pi (\theta |D_{n_{t}}) & \propto \prod_{i=1}^{n_{t}}p(Y_{i}|A_{i},X_{i},\theta )p(\theta ) \end{array}$$

In this article, the decision rule used at an interim or final analysis is defined with respect to a posterior probability statement about a marginal estimand γ(θ) , presented in equation (1), which is the average treatment effect in a population of interest. We note this marginal estimand can be defined as a contrast of population average quantities, such as a difference of means or a ratio of population-level event or survival probabilities. Let μ(θ;A) be the population level parameter for treatment group A used as input for contrast f(⋅) . Then γ(θ) can be represented by:

γ(θ)=f(μ(θ;A=1),μ(θ;A=0))

Unadjusted analyses yield posterior samples from the marginal parameter μ(θ;A) directly:

$$\mu (\theta ;A)=g^{-1}(\eta (\theta ;A))$$

Adjusted analyses, however, yield posterior samples from the conditional parameter μ(θ;A,X) for fixed covariate pattern X :

$$\mu (\theta ;A,X)=g^{-1}(\eta (\theta ;A,X,Z))$$

When a treatment effect is *collapsible*, it can be represented as a contrast between either the marginal or conditional parameters for a fixed covariate pattern X :

$$\begin{array}{rl} \gamma (\theta ) & =f(\mu (\theta ;A=1),\mu (\theta ;A=0))\\ & =f(\mu (\theta ;A=1,X),\mu (\theta ;A=0,X)) \end{array}$$

Thus, under collapsibility, samples from the posterior distribution of the marginal estimand can be obtained from either an unadjusted or adjusted analysis. When a treatment effect is *non-collapsible*, the marginal treatment effect cannot be represented as a contrast between conditional parameters for a fixed covariate pattern X .^16^ This is commonly the case for treatment effects modeled by GLMs or in the presence of treatment–covariate interactions. As an example, consider a hypothetical RCT with a binary endpoint that follows the following logistic regression model with binary treatment assignment A and binary covariate X , where P(X=1)=P(A=1)=0.5 and where ϕ=log(5) , β=log(10) , and θ={ϕ,β} :

logit(P(Y=1∣A,X))=ϕA+βX

Define μ(θ;A,X) to be the treatment-specific conditional risk. The conditional odds ratio for those who are treated versus untreated can be represented as a contrast of these conditional risk parameters and is 5 regardless of the value of X . To find the marginal odds ratio, the treatment-specific conditional risks must be averaged with respect to the distribution of X before calculating the odds ratio, effectively collapsing over X in a stratified two-by-two table. Doing so gives treatment-specific marginal risks μ(θ;A), which are used to obtain a marginal odds ratio of 4.1. This value is smaller than that for the conditional odds ratio and shows the marginal odds ratio cannot be represented as a contrast of the treatment-specific conditional risks. Thus, the odds ratio is non-collapsible (see Section G of the Online Supplemental Material for more details). Under non-collapsibility, samples from the posterior distribution of the marginal estimand can still be obtained directly from unadjusted analyses, but not from adjusted analyses. To obtain samples from the posterior distribution of the marginal estimand using an adjusted analysis, the posterior samples of μ(θ;A,X) must be marginalized with respect to the distribution of X , yielding samples of μ(θ;A), which are then used in the contrast:

$$\mu (\theta ;A)=\int_{X}\mu (\theta ;A,X)p(X)dX$$

This marginalization is commonly called standardization or Bayesian G-computation for point treatments.^16 –22^ The integral over p(X) is approximated through summation where, for each of s=1,…,S Monte Carlo samples $\theta_{s}$ from $\pi (\theta |D_{n_{t}})$ , the following calculation is performed:

(2) $$\mu (\theta_{s};A)\approx \sum_{i=1}^{n_{t}}w_{i,s}\mu (\theta_{s};A,x_{i})$$

The $x_{i}$ are the covariate patterns used for adjustment and which are contained in the joint empirical distribution of the collected sample data. The weights $w_{s}=(w_{1,s},\ldots ,w_{n_{t},s})$ are sampled as $w_{s}∼\text{Dirichlet}(1_{n_{t}})$ , corresponding to the Bayesian bootstrap, where $1_{n_{t}}$ is the $n_{t}$ -dimensional vector of 1 ’s.^23–25^ This can then be used to obtain a single sample from the posterior of the marginal treatment effect:

$$\gamma (\theta_{s})=f(\mu (\theta_{s};A=1),\mu (\theta_{s};A=0))$$

Note that when any $X_{j}$ for j=1,…,p is the propensity score being jointly modeled with the outcome of interest, a different Bayesian bootstrap procedure should be utilized.^26^ For the remainder of the article, it is assumed X does not contain a propensity score estimated in this manner.

The procedure above enables S samples from the posterior distribution of the marginal estimand γ(θ) to be obtained using an arbitrary adjustment model. This allows for a direct performance comparison between adjustment models within Bayesian adaptive designs. Below we describe this procedure within the context of specific models that are most commonly used for different outcome types in clinical trials.

### 3.1. Collapsible treatment effects

#### 3.1.1. Difference in means: No treatment–covariate interactions

Consider the difference in means of a continuous endpoint under the assumption of no treatment–covariate interactions (i.e. Z=∅ ; homogeneity of the treatment effect),

γ(θ):=μ(θ;A=1)−μ(θ;A=0)

where μ(θ;A=a)=E[Y∣A=a;θ] . This marginal estimand represents the difference in expected outcomes between those assigned to treatment versus those assigned to control. Estimation proceeds assuming independent outcomes and the following model:

$$\begin{array}{rl} & p(Y_{i}|A_{i},X_{i},\theta )=\text{Normal}(\mu (\theta ;A_{i},X_{i}),\sigma^{2})\\ & \mu (\theta ;A_{i},X_{i})=\beta_{0}+\phi A_{i}+X_{i}\beta \\ & \theta =\{\beta_{0},\phi ,\beta ,\sigma^{2}\} \end{array}$$

Since this treatment effect is collapsible, posterior samples of the marginal estimand can be obtained from either an unadjusted or adjusted analysis, and samples from the posterior distribution of the treatment indicator coefficient ϕ are commonly used.

### 3.2. Non-collapsible treatment effects

#### 3.2.1. Difference in means: Treatment–covariate interactions

Consider again a continuous outcome, but under the assumption of at least one treatment–covariate interaction (i.e. Z≠∅ ; treatment effect heterogeneity). The difference in means is non-collapsible. Estimation proceeds assuming independent outcomes and the following model:

$$\begin{array}{rl} & p(Y_{i}|A_{i},X_{i},\theta )=\text{Normal}(\mu (\theta ;A_{i},X_{i}),\sigma^{2})\\ & \mu (\theta ;A_{i},X_{i})=\beta_{0}+\phi A_{i}+X_{i}\beta +(A_{i}\cdot Z_{i})\omega \\ & \theta =\{\beta_{0},\phi ,\beta ,\omega ,\sigma^{2}\} \end{array}$$

Since this estimand is non-collapsible, posterior samples of the conditional μ(θ;A,X) must be marginalized using (2) before forming the contrast to obtain a posterior sample of the marginal difference in means. An outline of this procedure is provided in Appendix A in the Online Supplemental Materials.

#### 3.2.2. Relative risk and odds ratio

Consider a dichotomous outcome which is modeled as a Bernoulli random variable. Examples of commonly used marginal estimands include the relative risk

γ(θ):=μ(θ;A=1)/μ(θ;A=0)

and the odds ratio

$$\gamma (\theta ):=\frac{\mu (\theta ;A=1)/(1-\mu (\theta ;A=1))}{\mu (\theta ;A=0)/(1-\mu (\theta ;A=0))}$$

where μ(θ;A=a)=E[Y∣A=a;θ] . The relative risk represents the ratio comparing the risk of an event for those assigned to treatment versus those assigned to the control. The odds ratio represents the ratio comparing the odds of an event for those assigned to treatment versus those assigned to control. Estimation proceeds assuming independent outcomes and the following model:

$$\begin{array}{rl} & p(Y_{i}|A_{i},X_{i},\theta )=\text{Bernoulli}(\mu (\theta ;A_{i},X_{i}))\\ & \mu (\theta ;A_{i},X_{i})={\text{logit}}^{-1}(\beta_{0}+\phi A_{i}+X_{i}\beta +(A_{i}\cdot Z_{i})\omega )\\ & \theta =\{\beta_{0},\phi ,\beta ,\omega \} \end{array}$$

To obtain posterior samples of the marginal relative risk or odds ratio, posterior samples of the conditional μ(θ;A,X) must be marginalized using (2) before forming the contrast, an outline of which is provided in Appendix A in the Online Supplemental Materials.

#### 3.2.3. Hazard ratio

Let T denote the time to an event of interest. Let h(t∣A) represent the hazard, the instantaneous event rate at time t , for those assigned to treatment A :

$$h(t|A)=\lim_{\Delta t\to 0}\frac{P(t\leq T<t+\Delta t|T>t,A)}{\Delta t}$$

Under the assumption of no competing risks, there is a one-to-one relationship between the hazard and survival probability at time t :

$$S(t|A)=\exp\;\left(-\int_{v=0}^{t}h(v|A)dv\right)\;$$

Further assuming proportional hazards, an estimand of interest is the marginal hazard ratio

γ(θ):=log{μ(θ;A=1)}/log{μ(θ;A=0)}

where μ(θ;A=a)=S(t∣A=a;θ) . This estimand represents the ratio comparing the hazard of those assigned to treatment versus those assigned to control. We note that the estimation framework described below is general and may be utilized to target other estimands of interest (e.g. the risk difference or risk ratio).

Under an RCT framework, which allows for right censoring only, the time from a participant’s initial enrollment to an event of interest may occur after the trial has ended. Let $T_{i}$ be the ith participant’s observed event time or right censoring time. Let $\delta_{i}$ be the ith participant’s observation indicator, where $\delta_{i}=1$ means the event time is observed before the end of the trial and where $\delta_{i}=0$ means the event time is right censored. Let $Y_{i}=\{T_{i},\delta_{i}\}$ be the observed data. On the hazard scale, the hazard of the event at time t for the ith participant can be modeled as below, where $h_{0}(t)$ is the baseline hazard function:

$$\begin{array}{rl} h_{i}(t|A_{i},X_{i}) & =h_{0}(t)\exp\;(\eta_{i})\\ \eta_{i} & =\phi A_{i}+X_{i}\beta +(A_{i}\cdot Z_{i})\omega \end{array}$$

This yields the corresponding survival probability:

$$\begin{array}{rl} S_{i}(t|A_{i},X_{i}) & =\exp\;\left(-\int_{v=0}^{t}h_{i}(v|A_{i},X_{i})dv\right)\\ & =\exp\;\left(-\int_{v=0}^{t}h_{0}(v)\exp\;(\eta_{i})dv\right) \end{array}$$

The baseline hazard function may be flexibly modeled, with one possible choice being through M-splines.^27^ Let M(t;ψ,k,δ) be an M-spline function:

$$M(t;\psi ,k,\delta )=\sum_{l=1}^{L}\psi_{l}M_{l}(t;k,\delta )$$

Here ψ is the vector of coefficients for the L M-spline basis terms, with degree δ and knot locations k . Integrating this M-spline function yields the following I-spline function, which is evaluated using the same coefficients, degrees, and knot locations:

$$I(t;\psi ,k,\delta )=\sum_{l=1}^{L}\psi_{l}I_{l}(t;k,\delta )$$

Both M-spline and I-spline functions can be evaluated analytically.^28^ By flexibly modeling the baseline hazard with M-splines, the hazard and the survival probability become, respectively:

$$\begin{array}{rl} h_{i}(t|A_{i},X_{i}) & =M(t;\psi ,k,\delta )\exp\;(\eta_{i})\\ S_{i}(t|A_{i},X_{i}) & =\exp\;\left(-\int_{v=0}^{t}M(v;\psi ,k,\delta )\exp\;(\eta_{i})dv\right)\\ & =\exp\;\left(-I(t;\psi ,k,\delta )\exp\;(\eta_{i})\right) \end{array}$$

Estimation then proceeds by assuming independent outcomes and the following model:

$$\begin{array}{rl} & p(Y_{i}|A_{i},X_{i},\theta )=S_{i}(T_{i}|A_{i},X_{i}{)}^{1-\delta_{i}}h_{i}(T_{i}|A_{i},X_{i}{)}^{\delta_{i}}\\ & \theta =\{\psi ,\phi ,\beta ,\omega \} \end{array}$$

Posterior samples of the marginal hazard ratio are obtained by first marginalizing samples of μ(θ;A,X)=S(t∣A=a,X;θ) using (2) and then forming the contrast.^21,29,30^ This procedure is outlined in Appendix A in the Online Supplemental Materials.

## 4. Simulation study

In this section, we perform simulations for the design and models described in the previous sections. We consider a design with a superiority-stopping rule where superiority is declared at any interim or final analysis performed at time t if $T(D_{n_{t}})>0.99$ . The same value of u=0.99 is selected for all maximum sample sizes (max ss) to control the overall T1E of the unadjusted model (i.e. T1E below 0.05). The unadjusted model is selected as a conservative choice for trial planning purposes, since the true strength of any covariate effects and adjustment benefit may not be known in practice at the trial planning stage.^3^ Note that our interest is in comparing the performance of different adjustment models, not optimizing u for each max ss and model, so a single conservative value of u above is chosen for all simulations. Our marginal treatment effects of interest are the difference in means of a normal endpoint under the assumption of no treatment–covariate interactions, the relative risk under a binary endpoint, and the hazard ratio under a time-to-event endpoint. Data-generating processes with five covariates and a treatment assignment indicator are used for multiple sample sizes with each endpoint. We consider adjustment models which include several forms of misspecification and which incorporate varying levels of prior information for the covariate effects. To obtain marginal estimates from the adjustment models, the procedures described in the previous section are utilized. We follow a setup similar to that reported in a previous study which investigated covariate adjustment for endpoints commonly used in COVID-19 RCTs^3^: for each max ss with each endpoint, three treatment effect values are chosen. The first is the null treatment effect, and the second and third are those where the unadjusted model achieves roughly 50% and 80% power. This excludes the scenarios of a max ss of 100 under the binary and time-to-event endpoints, whose second and third treatment effect sizes are chosen as those where the unadjusted model achieves roughly 30% or 40% power. This ensures all simulations maintain realistic values for the marginal treatment effects, and that the impact of covariate adjustment is compared at a value of the treatment effect for which trials are commonly powered (i.e. 80%). For each max ss with each endpoint, the impact of covariate adjustment is quantified through the values of the following design operating characteristics: power, T1E, expected sample size, probability of stopping early, bias, and root mean squared error (RMSE).

### 4.1. Data generating mechanisms

For each combination of endpoint and max ss {100,200,500,1000} , the data-generating mechanisms for the treatment assignment and covariate distributions measured at baseline are shown below, where joint independence between all variables is assumed. Letting η represent the linear predictor used in the data-generating mechanisms, the set {β,ϕ} represents the conditional covariate and treatment effects on the linear predictor scale:

$$\begin{array}{rl} & \eta =\beta_{0}+\phi A+\beta_{1}X_{1}+\beta_{2}X_{2}+\beta_{3}X_{3}+\beta_{4}X_{3}^{2}+\beta_{5}X_{5}\\ & \{A,X_{1},X_{2},X_{6}\}∼\text{Bernoulli}(0.5)\\ & \{X_{3},X_{5},X_{7},X_{8}\}∼\text{Normal}(0,1)\\ & \beta =(\beta_{0},\beta_{1},\beta_{2},\beta_{3},\beta_{4},\beta_{5})\\ & \gamma_{\text{max ss}}=S(\eta )\\ & \Theta =\{(\gamma_{100}\times \beta ),(\gamma_{200}\times \beta ),(\gamma_{500}\times \beta ),(\gamma_{1000}\times \beta )\} \end{array}$$

Note that the variables $\left\{X_{6},X_{7},X_{8}\right\}$ are noise and are not predictive of, or correlated with, any other variables in the data-generating mechanism. For the binary endpoint, $\beta_{0}$ is optimized to generate datasets that exhibit the correct marginal control event risk of $p_{\mathrm{ctr}}=0.3$ (Appendix B in the Online Supplemental Materials). For the continuous and time-to-event endpoints, $\beta_{0}=0$ . For the time-to-event endpoint, an exponential baseline hazard with rate λ=0.02 is used. For the non-collapsible treatment effects, the true values of the marginal estimand γ=γ(θ)=f(μ(θ;A=1),μ(θ;A=0)) do not equal the conditional treatment effects ϕ=f(μ(θ;A=1,X),μ(θ;A=0,X)) for fixed X . Thus, the reported values of the marginal estimands are obtained through simulation (denoted by S(⋅) ; Appendix B in the Online Supplemental Materials), and the values of γ and ϕ are reported together. For the continuous endpoint, β=(0,0.5,−0.25,0.5,−0.05,0.25) . For the binary endpoint, β=(−1.26,1,−0.5,1,−0.1,0.5) . For the time-to-event endpoint, β=(0,1,−0.5,1,−0.1,0.5) . For each max ss within each outcome type, 1000 treatment–covariate datasets are generated. These are used to generate 1000 different outcome vectors for each value of the marginal treatment effect within the corresponding max ss. The specific parameter values used for all simulations are included in Table 1.

**Table 1.** Simulation study parameter settings for the marginal estimand ( γ ) and conditional treatment effect ( ϕ ) for each endpoint and maximum sample size (max ss).

Endpoint	Parameter settings					
	max ss	γ	max ss	γ
Continuous	100	0	500	0
	100	−0.52	500	−0.22
	100	−0.73	500	−0.32
	200	0	1000	0
	200	−0.36	1000	−0.16
	200	−0.52	1000	−0.22
	max ss	γ	ϕ	max ss	γ	ϕ
Binary	100	1	0	500	1	0
	100	0.53	−0.99	500	0.72	−0.56
	100	0.46	−1.21	500	0.60	−0.82
	200	1	0	1000	1	0
	200	0.59	−0.86	1000	0.80	−0.39
	200	0.41	−1.36	1000	0.72	−0.54
	max ss	γ	ϕ	max ss	γ	ϕ
Time-to-event	100	1	0	500	1	0
	100	0.65	−0.68	500	0.78	−0.39
	100	0.60	−0.79	500	0.71	−0.54
	200	1	0	1000	1	0
	200	0.69	−0.59	1000	0.85	−0.27
	200	0.57	−0.86	1000	0.78	−0.39

The marginal estimands for the continuous, binary, and time-to-event are, respectively, the difference in means under the assumption of no treatment–covariate interactions, the relative risk, and the hazard ratio.

### 4.2. Adjustment models

Six adjustment models are considered for all endpoints:

1. Correct:

$\beta_{0}+\phi A+\beta_{1}X_{1}+\beta_{2}X_{2}+\beta_{3}X_{3}+\beta_{4}X_{3}^{2}+\beta_{5}X_{5}$

2. No quad:

$\beta_{0}+\phi A+\beta_{1}X_{1}+\beta_{2}X_{2}+\beta_{3}X_{3}+\beta_{5}X_{5}$

3. Correct noise:

$\beta_{0}+\phi A+\beta_{1}X_{1}+\beta_{2}X_{2}+\beta_{3}X_{3}+\beta_{4}X_{3}^{2}+\beta_{5}X_{5}+\beta_{6}X_{6}+\beta_{7}X_{7}+\beta_{8}X_{8}$

4. Correct prior:

$\beta_{0}+\phi A+\beta_{1}^{†}X_{1}+\beta_{2}^{†}X_{2}+\beta_{3}^{†}X_{3}+\beta_{4}^{†}X_{3}^{2}+\beta_{5}^{†}X_{5}$

5. Correct strong prior:

$\beta_{0}+\phi A+\beta_{1}^{††}X_{1}+\beta_{2}^{††}X_{2}+\beta_{3}^{††}X_{3}+\beta_{4}^{††}X_{3}^{2}+\beta_{5}^{††}X_{5}$

6. Unadjusted:
$\beta_{0}+\phi A$ .

The *correct* model corresponds to an adjustment model which matches the data-generating mechanism. The *no quad* model drops the quadratic component of $X_{3}$ from the *correct* model. The *correct noise* model adds noise variables $\left\{X_{6},X_{7},X_{8}\right\}$ to the *correct* model. These three models include priors for all parameters which are weakly informative only. The *correct prior* model is the same as the *correct* model, but includes priors for the covariate effects centered at the values used in the data-generating mechanism. Similarly, the *correct strong prior* model both centers and re-scales these priors to be more informative. Note that the prior for the treatment indicator coefficient remains weakly informative in these models. Finally, the *unadjusted* model includes only the binary treatment indicator and uses weakly informative priors.

All simulations are performed using R (version 4.2.1). All modeling is performed using the GLM and survival functionality of the rstanarm package (version 2.21.2), a front-end to the STAN probabilistic programming language.^27,31,32^ For all coefficients other than $\left\{\beta_{j}^{†},\beta_{j}^{††}\right\}$ , the package’s default weakly informative priors are used.^33^ These priors induce moderate regularization and help improve computational stability. The prior for the intercept is placed after all covariates have been internally centered by rstanarm.

Under a normal likelihood for the continuous endpoint, this equates to the following priors, where $\beta_{0,c}$ represents the intercept’s prior after covariate centering has been performed:

$$\begin{array}{r} \begin{array}{rlrl} & \beta_{0,c}∼\text{Normal}(\bar{y},2.5s_{y}) & & \beta_{j}^{†}∼\text{Normal}(\beta_{j},2.5(s_{y}/s_{x}))\\ & \phi ∼\text{Normal}(0,2.5(s_{y}/s_{a})) & & \beta_{j}^{††}∼\text{Normal}(\beta_{j},s_{y}/s_{x})\\ & \beta_{j}∼\text{Normal}(0,2.5(s_{y}/s_{x})) & & \sigma ∼\text{Exponential}(1/s_{y}) \end{array} \end{array}$$

Under the binary and time-to-event endpoints, the above priors for {ϕ,β} are used with $\bar{y}=0$ and $s_{y}=1$ . For the time-to-event endpoint, the coefficients of the M-spline basis ( ψ ) are constrained to a simplex to ensure the identifiability of both the basis and linear predictor intercepts. Thus, the basis coefficients receive rstanarm’s default Dirichlet prior.^27^ All models specify three Markov chains, each with 2000 posterior samples. Half of the samples within each chain are used during the warm-up period, so 3000 posterior samples in total are available for inference. Given the scale of the simulations performed, visual diagnostics assessing convergence of the Markov chains are not performed. Rather, for all simulations, values of STAN’s implementation of the Gelman–Rubin $\hat{R}$ statistic are assessed to ensure Markov chain convergence.^34^

The following null and alternative hypotheses are specified for the continuous, binary, and time-to-event (TTE) endpoints, where γ(θ) is the marginal difference in means, marginal relative risk, and marginal hazard ratio, respectively:

$$\begin{array}{rl} \text{Continuous: } & H_{0}:\gamma (\theta )\geq 0\;\text{versus}\;H_{A}:\gamma (\theta )<0\\ \text{Binary and TTE: } & H_{0}:\gamma (\theta )\geq 1\;\text{versus}\;H_{A}:\gamma (\theta )<1 \end{array}$$

For the continuous and binary endpoints, all outcomes are assumed to be observed immediately upon participant enrollment. For the time-to-event endpoint, it is assumed all outcomes are observed strictly after enrollment. For the continuous endpoint, interim analyses are performed after every 25, 50, 125, and 250 participants are enrolled for maximum sample sizes of 100, 200, 500, and 1000, respectively. For the binary and time-to-event endpoints, interim analyses are event-driven. For the binary endpoint, interim analyses are performed after at least 10, 20, 50, and 100 new events occur for max ss 100, 200, 500, and 1000, respectively. For the time-to-event endpoint, interim analyses are performed after at least 20, 40, 100, and 200 new events occur for max ss 100, 200, 500, and 1000, respectively. These numbers are chosen for each endpoint to ensure that on average the total number of analyses performed under the null treatment effect is less than four, which helps control the T1E. They are also large enough to ensure there is a moderate chance of stopping at an early interim analysis under the non-null treatment effects. For the continuous and binary endpoints, interim analyses are performed until the trial is stopped early for superiority or until the max ss is reached, at which time the final analysis is performed. For the time-to-event endpoint, interim analyses are performed until the trial is stopped early for superiority or until 50 time units from the start of the trial are reached, at which time the final analysis is performed. For this endpoint, participant enrollment is permitted until 25 time units. This ensures that participants enrolled at later time points are under follow-up long enough to have a moderately high probability of experiencing the event before the end of the trial. It also ensures that the maximum number of participants will be enrolled if there is not clear evidence of superiority at an early interim analysis. No loss to follow-up is assumed and administrative censoring of those still at risk is performed at 50 time units from the start of the trial.

### 4.3. Simulation study results

Within each outcome type and max ss, the following design operating characteristics are investigated: power, T1E, probability of stopping early, expected sample size, posterior median bias, and RMSE. Since the sampling distribution of the test statistic $T(D_{n_{t}})$ is unknown, power and the T1E are estimated via Monte Carlo using the 1000 datasets. While this number is lower than that required by the FDA for adaptive simulations used in RCTs,^35^ our goal here is to compare model adjustment performance, not to obtain precise estimates of operating characteristics. The probability of stopping early is estimated as the proportion of times the trial stops before performing a final analysis. In the continuous and binary outcomes, this is the proportion of times the trial stops before enrolling the maximum number of participants. In the time-to-event outcome, this is the proportion of times the trial stops before 50 time units. In Bayesian adaptive designs which allow for early stopping, sample size is a random variable. Thus, the expected sample size is estimated as the average of the 1000 sample sizes at the trial end. Posterior median bias is defined as the bias resulting from using the posterior median $\hat{\gamma }$ obtained from an adjustment model as an estimator for the value of γ used in the simulation and is estimated through Monte Carlo using the 1000 datasets. The Monte Carlo distribution of RMSE is displayed for each value of the marginal estimand. Here the entire posterior distribution from an adjustment model is used as the estimator for γ , so this is equivalent to the posterior expected squared error loss. For each of the 1000 simulations, a single value of RMSE is obtained using the 3000 posterior draws $\gamma_{s}$ for the value of γ used in the simulation:

$$\text{RMSE}=\sqrt{\frac{1}{3000}\sum_{s=1}^{3000}(\gamma_{s}-\gamma {)}^{2}}$$

**Figure 1.**
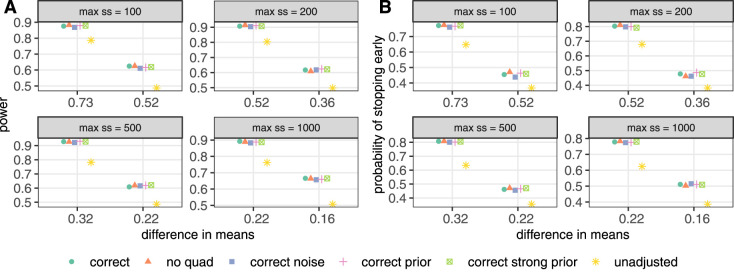
Continuous outcome: (A) Power and (B) probability of stopping early. Panels correspond to various maximum sample sizes (max ss). Points are jittered horizontally.

**Figure 2.**
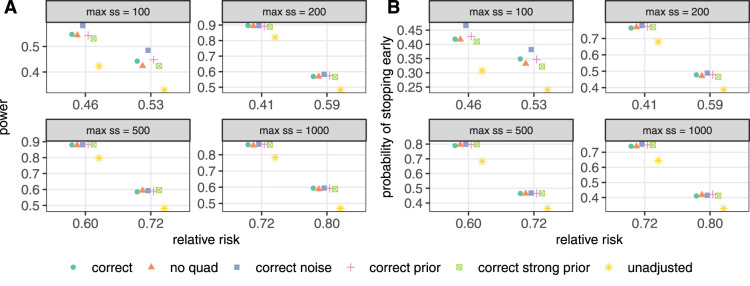
Binary outcome. (A) Power and (B) probability of stopping early. Panels correspond to various maximum sample sizes (max ss). Points are jittered horizontally.

**Figure 3.**
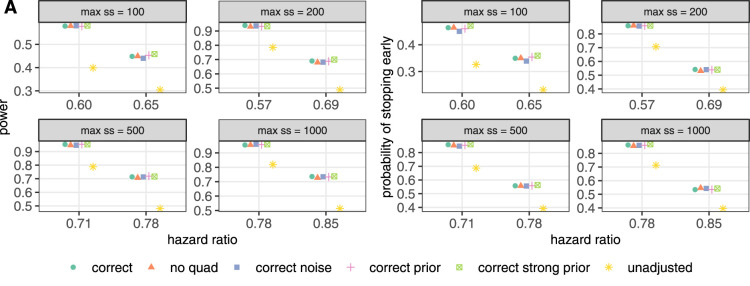
Time-to-event outcome. (A) Power and (B) probability of stopping early. Panels correspond to various maximum sample sizes (max ss). Points are jittered horizontally.

Results for the continuous, binary, and time-to-event endpoints are displayed in Figures 1 to 3 and Tables 2 to 4. For all endpoints, adjusting for variables known to be associated with the outcome increases the power of the trial and the probability of stopping the trial early as compared to the unadjusted analysis (Figures 1 to 3). Additionally, failing to correctly specify the functional form of a covariate (*no quad*) has only a minor impact on power and the probability of stopping early. Under all scenarios, incorporating stronger prior information appears to provide little to no benefit as compared to the weakly informative priors used in the *correct* models. This results from the priors being dominated by the data due to the high effective sample sizes. For the binary endpoint, this is induced by the control event risk of 0.3, which ensures that a moderately large number of events occurs throughout the trial. For the time-to-event endpoint, this is induced by the exponential baseline hazard rate of λ=0.02 , which ensures there are a large number of events within the maximum time limit of 50 time units.

**Table 2.** Continuous outcome.

				Expected sample size		
	Adjustment model	T1E	${\text{Bias}}^{*}$	γ=0	γ=−0.52	γ=−0.73
max ss = 100	Correct	0.034	−0.013	98.3	78.6	62.5
	No quad	0.037	−0.015	98.2	78.0	62.5
	Correct noise	0.031	−0.012	98.6	80.3	64.8
	Correct prior	0.032	−0.012	98.5	78.3	62.9
	Correct strong prior	0.033	−0.012	98.3	78.4	62.5
	Unadjusted	0.028	−0.007	98.5	82.6	68.9
				γ=0	γ=−0.36	γ=−0.52
max ss = 200	Correct	0.039	−0.011	196.8	154.7	116.0
	No quad	0.032	−0.009	197.5	156.2	115.4
	Correct noise	0.036	−0.012	196.9	156.2	118.7
	Correct prior	0.043	−0.012	196.4	154.5	116.3
	Correct strong prior	0.041	−0.012	196.6	155.0	116.8
	Unadjusted	0.039	−0.017	196.5	163.1	130.7
				γ=0	γ=−0.22	γ=−0.32
max ss = 500	Correct	0.036	−0.008	491.1	389.1	290.4
	No quad	0.031	−0.007	493.0	388.8	291.4
	Correct noise	0.032	−0.006	492.8	391.1	293.0
	Correct prior	0.032	−0.007	492.8	388.5	293.8
	Correct strong prior	0.032	−0.007	492.8	388.8	291.0
	Unadjusted	0.032	−0.006	492.4	415.6	343.1
				γ=0	γ=−0.16	γ=−0.22
max ss = 1000	Correct	0.031	−0.003	987.0	753.0	598.8
	No quad	0.028	−0.003	987.8	755.2	597.5
	Correct noise	0.030	−0.003	987.8	753.2	599.0
	Correct prior	0.031	−0.003	987.2	753.5	598.0
	Correct strong prior	0.029	−0.003	986.8	754.8	599.0
	Unadjusted	0.028	−0.002	989.0	817.8	690.2

T1E: Type 1 error rate; max ss: maximum sample size. T1E, bias under the null ( ${\text{Bias}}^{*}$ ), and expected sample size at three different values of the marginal difference in means ( γ ) for different max ss.

**Table 3.** Binary outcome.

				Expected sample size		
	Adjustment model	T1E	${\text{Bias}}^{*}$	γ=1	γ=0.53	γ=0.46
max ss = 100	Correct	0.063	0.031	97.0	86.3	83.6
	No quad	0.053	0.035	97.4	86.9	83.7
	Correct noise	0.079	0.031	96.2	84.2	81.0
	Correct prior	0.060	0.033	97.2	86.3	82.9
	Correct strong prior	0.052	0.035	97.7	88.1	84.5
	Unadjusted	0.034	0.058	98.6	90.7	88.3
				γ=1	γ=0.59	γ=0.41
max ss = 200	Correct	0.036	0.021	196.8	162.5	138.3
	No quad	0.031	0.023	197.6	164.0	138.6
	Correct noise	0.044	0.021	196.0	161.3	137.4
	Correct prior	0.036	0.021	196.5	162.9	138.0
	Correct strong prior	0.036	0.022	196.9	164.6	138.2
	Unadjusted	0.031	0.025	198.0	171.4	147.2
				γ=1	γ=0.72	γ=0.60
max ss = 500	Correct	0.028	0.010	493.7	404.9	334.9
	No quad	0.028	0.009	494.0	406.1	335.2
	Correct noise	0.031	0.010	493.9	402.7	334.8
	Correct prior	0.028	0.010	494.3	405.0	336.1
	Correct strong prior	0.027	0.010	494.2	404.7	337.2
	Unadjusted	0.026	0.016	494.6	426.0	367.0
				γ=1	γ=0.80	γ=0.72
max ss = 1000	Correct	0.022	0.008	992.0	823.2	681.5
	No quad	0.022	0.007	991.3	816.9	683.8
	Correct noise	0.022	0.007	991.4	816.4	677.3
	Correct prior	0.021	0.007	991.9	817.8	680.7
	Correct strong prior	0.021	0.008	992.6	821.0	677.3
	Unadjusted	0.024	0.010	990.2	859.6	734.2

T1E: Type 1 error rate; max ss: maximum sample sizes. T1E, bias under the null ( ${\text{Bias}}^{*}$ ), and expected sample size at three different values of the marginal relative risk ( γ ) for different max ss.

**Table 4.** Time-to-event outcome.

				Expected sample size		
	Adjustment model	T1E	${\text{Bias}}^{*}$	γ=1	γ=0.65	γ=0.60
max ss = 100	Correct	0.027	0.014	99.7	98.3	98.0
	No quad	0.027	0.017	99.7	98.3	97.9
	Correct noise	0.036	0.013	99.6	98.1	97.8
	Correct prior	0.029	0.013	99.6	98.3	97.9
	Correct strong prior	0.026	0.015	99.7	98.3	97.8
	Unadjusted	0.029	0.035	99.7	98.5	98.2
				γ=1	γ=0.69	γ=0.57
max ss = 200	Correct	0.033	0.000	199.4	193.1	189.4
	No quad	0.029	0.002	199.4	193.5	189.7
	Correct noise	0.030	−0.001	199.4	193.1	189.2
	Correct prior	0.031	0.000	199.4	193.1	189.3
	Correct strong prior	0.033	0.000	199.3	193.3	189.2
	Unadjusted	0.028	0.005	199.4	194.4	191.0
				γ=1	γ=0.78	γ=0.71
max ss = 500	Correct	0.026	0.003	498.4	477.9	464.8
	No quad	0.025	0.003	498.6	478.6	463.5
	Correct noise	0.027	0.003	498.6	477.6	464.2
	Correct prior	0.027	0.003	498.5	478.1	463.6
	Correct strong prior	0.026	0.003	498.4	477.8	463.2
	Unadjusted	0.022	0.011	498.6	483.4	470.6
				γ=1	γ=0.85	γ=0.78
max ss = 1000	Correct	0.026	0.001	996.9	955.4	912.4
	No quad	0.027	0.001	997.2	953.8	913.1
	Correct noise	0.022	0.001	997.4	953.3	910.7
	Correct prior	0.024	0.001	997.4	954.4	912.9
	Correct strong prior	0.026	0.001	997.4	955.4	911.9
	Unadjusted	0.019	0.003	997.9	962.8	929.6

T1E: Type 1 error rate; max ss: maximum sample sizes. T1E, bias under the null ( ${\text{Bias}}^{*}$ ), and expected sample size at three different values of the marginal hazard ratio ( γ ) for different max ss.

Compared to other adjustment models, adjusting for noise (*correct noise*) has minimal impact under most scenarios. However, for the smallest max ss under the binary endpoint (max ss = 100), adjustment for noise slightly increases power and the probability of stopping early as compared to the *correct* model. This may result from a non-negligible correlation being induced between the outcome and noise variables under this setting, and so adjustment provides a further power benefit. However, this comes at the cost of a strong inflation in the T1E as compared to the *correct* model (i.e. T1E=0.079 vs. T1E=0.063 in Table 3). This underscores the importance of adjusting only for variables that are known to be associated with the outcome and in a pre-specified manner.^36,37,6,9^

There is some suggestion that adjusted analyses tend to have slightly lower RMSE than unadjusted analyses under all scenarios. However, no clear pattern emerges for posterior median bias for the non-null treatment effects, where all adjustment models are comparable for practical purposes (Figures D.1 to D.3 in the Online Supplemental Materials). For all scenarios except the smallest max ss under the binary endpoint (max ss = 100), the posterior median bias for the non-null treatment effects is negative. This results from the estimated treatment effect being further from the null than the true treatment effect (i.e. overestimation), which is expected in trials which allow for early stopping for treatment superiority (i.e. truncated trials).^38–40^ For the null treatment effect, early stopping for superiority leads to non-zero but minimal bias under all endpoints and max ss (Tables 2 to 4). For the binary endpoint, it is consistently larger in magnitude for the *unadjusted* model. We note that bias under the null is negative for the continuous endpoint but positive for the binary and time-to-event endpoints. This results from the marginal difference in means being unbounded below, whereas the marginal relative risk and marginal hazard ratio are bounded below by zero. When bias under the null is evaluated for these latter estimands on the log scale, most values become negative as in the continuous endpoint case. We elaborate further on this overestimation-induced bias in Section F of the Online Supplemental Material.

Under all scenarios except the smallest max ss = 100 for the binary endpoint, the T1E is maintained below 0.05 for all adjustment models (Tables 2 to 4). Under the smallest max ss within the binary endpoint, however, all adjustment models lead to increased T1E as compared to the *unadjusted* model. This results from using too many covariates in the adjustment model given the low effective sample size, a phenomenon known as over-stratification.^7^ We observe that as the max ss, and thus effective sample size, increases, the inflation in the T1E disappears. Under the time-to-event endpoint, there is minimal inflation in the T1E for adjusted analyses as compared to the unadjusted analysis. This may also result from over-stratification as described above. Future work should determine the optimal number of covariates to include in an adjustment model under these scenarios to avoid over-stratification. Considering the substantial power gains achieved by adjusting under the time-to-event endpoint, and that the T1E is maintained by selecting a conservative value of the probability of superiority threshold u , this slight increase in the T1E is not likely to be problematic, however. Across all non-null treatment effect scenarios, the adjusted models have lower expected sample sizes than the unadjusted model. When combined with the probability of stopping early results, this implies that adjusted analyses are stopping more often and at earlier interim analyses for all endpoints. We note that the reduction in expected sample size is not as great for the time-to-event endpoint as compared to the other endpoints. This results from the max ss being included for any interim analyses conducted past the halfway point of the trial, since all trial participants are enrolled by this point. A final simulation (included in Section E of the Online Supplemental Material), which incorporated varying degrees of prior information on the treatment effect was performed for the binary endpoint. This resulted in increased power and probability of stopping early for smaller max ss, but at the cost of inflated Type 1 error. A more complete investigation of including informative priors on treatment effects remains as future work.

## 5. Application: CCEDRRN-ADAPT

In this section, we consider the design of a hypothetical platform trial that seeks to study the effectiveness of oral therapies against mild to moderate COVID-19 infection in individuals discharged from Canadian Emergency Departments. The trial design takes advantage of an already established network of physicians and researchers called the Canadian COVID-19 Emergency Department Rapid Response Network (CCEDRRN). We consider the first stage only, where a single oral therapy is compared to the standard of care. The binary outcome of interest is a composite endpoint of 28-day hospitalization or mortality. Realistic values used in the trial simulation performed below are taken from a COVID-19 Emergency Department risk model, developed by the CCEDRRN researchers.^41^ This risk model was developed using data from a high-quality, population-based registry, which was also developed by the CCEDRRN researchers.^42,43^ While the binary outcome for the risk model is all-cause emergency department and in-hospital mortality, this is very likely to be highly correlated with the trial’s composite outcome. Thus, the simulation’s results are expected to generalize to the composite endpoint as well.

The data-generating mechanism for the trial simulation is shown below, where a single max ss of 3000 is chosen due to the very low marginal control event risk ( $p_{\mathrm{ctr}}=0.07$ ) and reflects the sample size used in CCEDRRN-ADAPT. Letting Y=1 represent 28-day hospital admission or mortality, the marginal estimand of interest is the relative risk, γ(θ)=μ(θ;A=1)/μ(θ;A=0) where μ(θ;A=a)=E[Y|A=a;θ] . We assume γ(θ) is intention-to-treat, that is, we compare the outcome distributions of those who are assigned to treatment versus those who are assigned to control. As above, let η represent the linear predictor used in the data-generating mechanisms. The set {β,ϕ} represents the conditional covariate and treatment effects on the log-odds scale. As in the binary simulation case, the value of $\beta_{0}$ is optimized to ensure the generated datasets exhibit the correct marginal control event risk of $p_{\mathrm{ctr}}=0.07$ . The values of ϕ are selected as those where the unadjusted model achieves approximately 50% and 80% power. The value of γ=γ(θ) corresponding to a specific value of ϕ is determined through simulation, described in Appendix B of the Online Supplemental Materials. Thus, the values of ϕ and γ are reported together. Let F be a truncated normal distribution, parameterized by *post-truncation* values of its minimum ∧ , maximum ∨ , first quartile q1 , third quartile q3 , mean μ , and standard deviation σ . An algorithm to simulate from this distribution is provided in Appendix C of the Online Supplemental Materials.

The covariate distributions are simulated using values of summary statistics for the derivation cohort from a previously reported risk model for individuals presenting to Canadian Emergency Departments with COVID-19 symptoms.^41^ Covariates included in the risk model and whose summary statistics were available are included in the simulation. These include age in years ( $X_{1}$ ), respiratory rate upon arrival to the Emergency Department and measured in breaths/minute ( $X_{2}$ ), female sex ( $X_{3}$ ), chest pain ( $X_{4}$ ), and arrival from police or ambulance ( $X_{5}$ ). As the risk model was developed before COVID-19 vaccines were widely available, vaccination status is not included as a potential covariate. The corresponding risk model coefficients are used as the conditional effects $\left\{\beta_{1},\ldots ,\beta_{5}\right\}$ in the simulation. Since the summary statistics did not include covariance information, joint independence between all variables is assumed. Due to study inclusion criteria, the range of the distribution of age is set to be [18,90] . Due to biological constraints, the range of the distribution of respiratory rate is set to be [12,40] . Values for age and respiratory rate are simulated from the truncated normal distribution F described above.

A total of 1000 treatment–covariate datasets are generated, each containing 3000 participants. Using these same 1000 datasets, different outcome vectors are generated for each value of the marginal relative risk γ∈{1,0.73,0.63} , which corresponds to ϕ∈{0,−0.42,−0.60} . The outcome vectors are generated as follows:

$$\begin{array}{rl} & Y∼{\text{Bernoulli(logit}}^{-1}(\eta ))\\ & \eta =\beta_{0}+\phi A+\beta_{1}X_{1}+\beta_{2}X_{2}+\beta_{3}X_{3}+\beta_{4}X_{4}+\beta_{5}X_{5}\\ & X_{1}∼F(\wedge =18,\vee =90,q1=39,q3=70,\mu =54.7,\sigma =19.8)\\ & X_{2}∼F(\wedge =12,\vee =40,q1=18,q3=22,\mu =21,\sigma =6.2)\\ & X_{3}∼\text{Bernoulli}(0.478)\\ & X_{4}∼\text{Bernoulli}(0.216)\\ & X_{5}∼\text{Bernoulli}(0.403)\\ & p_{\mathrm{ctr}}=0.07\\ & \beta =(-10.76,0.092,0.097,-0.61,-0.80,0.63)\\ & \Theta =\{\gamma \times \beta \} \end{array}$$

The trial uses the same null and alternative hypotheses as those specified under the binary endpoint simulation above and includes a probability of superiority threshold of u=0.99 . Estimation of the marginal relative risk proceeds as previously described. Four adjustment models are considered and mirror those used in the binary simulations: *correct*, *correct prior*, *correct strong prior*, and *unadjusted*. All outcomes are assumed to be observed immediately upon participant enrollment. Interim analyses are event-driven, and 75 new events are required to be observed before performing an interim analysis. This ensures a moderately high probability of stopping at an earlier interim analysis given treatment superiority. Interim analyses continue until the trial is stopped early for superiority or until 3000 participants are enrolled, at which time the final analysis is performed.

**Figure 4.**
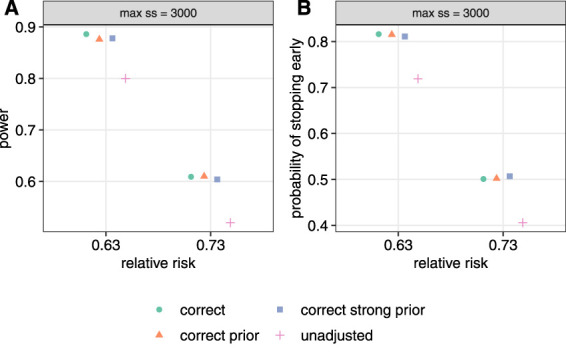
COVID-19 trial with binary outcome. (A) Power and (B) probability of stopping early. Points are jittered horizontally.

Results for the CCEDRRN-ADAPT design simulation study are summarized in Figure 4 and Table 5. Adjusting for variables known to be associated with the outcome increases the power of the trial and the probability of stopping the trial early as compared to the unadjusted analysis (Figure 4). As in the binary simulations above, including stronger priors on the covariate effects has minimal impact on power and the probability of stopping early as compared to the weakly informative priors used in the *correct* models. While there is some suggestion that adjusted analyses tend to have slightly lower RMSE than unadjusted analyses, all adjustment models have a comparable posterior median bias for the non-null treatment effects (Figure D.4 in the Online Supplemental Materials). The T1E is maintained below 0.05, and bias under the null is minimal, for all adjustment models (Table 5). As in the binary simulations above, there is slight inflation in bias under the null for the *unadjusted* model, however. The adjusted models have lower expected sample sizes than the unadjusted model across both non-null values of the relative risk. Again, we see the adjusted analyses are stopping more often and at earlier interim analyses as compared to the unadjusted analysis. For example, under a relative risk of 0.63, the *correct* model stops at the first interim analysis 50% of the time whereas the *unadjusted* model stops at the first interim analysis only 40% of the time.

**Table 5.** COVID-19 trial with binary outcome.

			Expected sample size		
Adjustment model	T1E	${\text{Bias}}^{*}$	γ=1	γ=0.73	γ=0.63
Correct	0.022	0.017	2969.7	2458.9	2047.3
Correct prior	0.022	0.017	2968.9	2455.2	2051.8
Correct strong prior	0.020	0.017	2971.5	2454.7	2056.0
Unadjusted	0.024	0.022	2972.7	2561.7	2220.9

T1E: Type 1 error rate. T1E, bias under the null ( ${\text{Bias}}^{*}$ ), and expected sample size at three different values of the marginal relative risk ( γ ).

## 6. Discussion

The impact of covariate adjustment and incorporation of prior information on covariate effects has not been previously investigated in Bayesian adaptive trials with early stopping criteria. In this article, we assessed this impact using a variety of adjustment models and incorporated varying levels of prior information or types of model misspecification. It was shown that covariate adjustment increases power and the probability of stopping early, and decreases expected sample size over all scenarios. Furthermore, adjusting for covariates leads to trials that stop more often and at earlier interim analyses, and can decrease RMSE as compared to unadjusted analyses. These findings are fairly robust to adjustment for noise variables, but extra caution is needed for small sample size trials (max ss = 100) with binary endpoints where noise adjustment may lead to inflated Type 1 error. This reinforces the existing best practice of only adjusting for covariates that have been pre-specified by subject matter experts.^36,37,6,9^ For the scenarios explored here, which had moderate effective sample sizes, weakly informative priors on the covariate effects perform well, with stronger prior information providing little benefit. This included the CCEDRRN-ADAPT COVID-19 RCT design. Although the control event risk (0.07) of this trial is small, the max ss was selected to be large enough to power the study for reasonable values of relative risk, which ensured moderately high effective sample sizes. Including stronger prior information is expected to be helpful in trials that have small effective sample sizes (e.g. oncology trials), so future work may consider covariate adjustment within these contexts. Although we did not consider designs that also include futility-stopping rules, we expect that the conclusions can be generalized.

In our simulation study, all covariates were assumed to be jointly independent. The assumption of independence may not hold in some cases and these should be investigated further. Since they carry similar information, adjusting for covariates that are moderately or highly correlated may yield smaller power increases than adjusting for approximately independent covariates. Future work might perform a simulation study to assess how different strengths of association between covariates impact the results reported here. Another limitation of the current work is that adjustment was shown under only a single set of covariate effects within each endpoint. There is strong evidence in favor of covariate adjustment when the covariate effects are known to be strong. However, previous simulations (not shown) showed that adjustment under weaker covariate effects yielded only modest benefits. This finding was especially pronounced in the case of the non-linear endpoints. Future work might investigate a wider range of covariate effects to ascertain the magnitude that is required for covariate adjustment to be more than just modestly beneficial, though this may be context-dependent.

It is important to note that the marginalization procedure used in this work is sensitive to the joint empirical distribution of the covariates with respect to which the conditional posterior samples are being marginalized. That is, the marginalization procedure is sensitive to the participants enrolled in the trial. While trialists work hard to obtain representative samples for use in RCTs, the participants volunteer and consent to be enrolled in the trials. Thus they are not a random sample from the population of interest.^44^ If the sample is not as representative of the population of interest as desired, indirect standardization could be performed. Here, conditional posterior samples would be marginalized with respect to a set of covariate patterns that more closely resembles the population of interest. Additionally, the participant covariate patterns could be augmented by *pseudo-participant* covariate patterns until the sample is more representative. These could be acquired from registry data or from participants in previous trials that contained a similar population and target of interest.

This work focused on Bayesian adaptive designs which employed simple randomization, where covariate adjustment takes place within an adaptive *decision* rule. Covariate adjustment may also occur within an adaptive *allocation* rule, such as in Covariate Adjusted Response Adaptive designs.^45,46^ These designs estimate treatment arm allocation probabilities from models that include covariates. While frequentist in nature, similar ideas can be applied within Bayesian response adaptive randomization designs. Since adaptive randomization leads to greater covariate imbalance across treatment arms, including covariate adjustment in both the adaptive decision and adaptive allocation rules may provide greater benefit than either do individually. This is an interesting direction for future work.

The simulation study above included variants of the *correct* adjustment model, which corresponded to the data-generating mechanism used. In reality, this will be unknown. Variable selection and shrinkage methods might be employed to select covariates to be used in the adjustment model. Particularly, Bayesian model averaging can be used where the decision rules would use marginal posterior samples obtained from a consensus of plausible models and might be less sensitive to any single adjustment model misspecification. However, this approach is likely to be very computationally intense.

Another interesting direction of future research is exploration of Bayesian nonparametric models, such as Gaussian processes, to consider more flexible functional forms that adjust for covariates. This approach might be especially advantageous in a setting where the underlying association between the adjustment covariates and outcome is complex and hard to correctly specify, which might include a high degree of covariate non-linearity or interaction.

## Supplemental Material

sj-pdf-1-smm-10.1177_09622802241227957 - Supplemental material for Covariate adjustment in Bayesian adaptive randomized controlled trials Supplemental material, sj-pdf-1-smm-10.1177_09622802241227957 for Covariate adjustment in Bayesian adaptive randomized controlled trials by James Willard, Shirin Golchi and Erica EM Moodie in Statistical Methods in Medical Research
