# Supplementary material for: Covariate adjustment in Bayesian adaptive randomized controlled trials
Source: Stat Methods Med Res. 2024 Feb 7;33(3):480–97. doi: 10.1177/09622802241227957 (PMC10981207; doi:10.1177/09622802241227957)
Supplement: sj-pdf-1-smm-10.1177_09622802241227957 - Supplemental material for Covariate adjustment in Bayesian adaptive randomized controlled trials [file sj-pdf-1-smm-10.1177_09622802241227957.pdf]

Covariate Adjustment in Bayesian Adaptive Randomized Controlled Trials: Online Supplementary Materials

James Willard, Shirin Golchi, and Erica EM Moodie

A Marginalization Procedures

A.1 Continuous Outcome: Difference in Means

Let Y be a Normally distributed outcome with the target of inference being the marginal difference in means:

$$\gamma(\boldsymbol{\theta}) := \mu(\boldsymbol{\theta}; A = 1) - \mu(\boldsymbol{\theta}; A = 0)$$

where $\mu(\boldsymbol{\theta}; A = a) = E[Y \mid A = a; \boldsymbol{\theta}]$. Under the assumption of at least one treatment-covariate interaction (i.e., $\mathbf{Z} \neq \emptyset$; treatment effect heterogeneity), the difference in means is non-collapsible. Estimation proceeds assuming independent outcomes and the following model:

$$p(Y_i \mid A_i, \mathbf{X}_i, \boldsymbol{\theta}) = \text{Normal}(\mu(\boldsymbol{\theta}; A_i, \mathbf{X}_i), \sigma^2) \qquad \mathcal{L} = \prod_{i=1}^{n_t} p(Y_i \mid A_i, \mathbf{X}_i, \boldsymbol{\theta})$$

$$\mu(\boldsymbol{\theta}; A_i, \mathbf{X}_i) = \beta_0 + \phi A_i + \mathbf{X_i}\boldsymbol{\beta} + (A_i \cdot \mathbf{Z_i})\boldsymbol{\omega}$$

$$\boldsymbol{\theta} = \{\beta_0, \phi, \boldsymbol{\beta}, \boldsymbol{\omega}, \sigma^2\} \qquad \pi(\boldsymbol{\theta} \mid \mathcal{D}_{n_t}) \propto \prod_{i=1}^{n_t} p(Y_i \mid A_i, \mathbf{X}_i, \boldsymbol{\theta}) p(\boldsymbol{\theta}).$$

Samples from the posterior distribution of $\gamma(\boldsymbol{\theta})$ are obtained from adjusted analyses by marginalizing $s = 1, ..., S$ samples of the conditional $\mu(\boldsymbol{\theta}; A, \mathbf{X})$ before forming the contrast:

$$\begin{aligned}
\gamma(\boldsymbol{\theta}_s) &= \mu(\boldsymbol{\theta}_s; A = 1) - \mu(\boldsymbol{\theta}_s; A = 0) \\
&= \int_{\mathbf{X}} \mu(\boldsymbol{\theta}_s; A = 1, \mathbf{X}) p(\mathbf{X}) d\mathbf{X} - \int_{\mathbf{X}} \mu(\boldsymbol{\theta}_s; A = 0, \mathbf{X}) p(\mathbf{X}) d\mathbf{X} \\
&= \int_{\mathbf{X}} (\beta_{0,s} + \phi_s + \mathbf{X}\boldsymbol{\beta}_s + \mathbf{Z}\boldsymbol{\omega}_s) p(\mathbf{X}) d\mathbf{X} - \int_{\mathbf{X}} (\beta_{0,s} + \mathbf{X}\boldsymbol{\beta}_s) p(\mathbf{X}) d\mathbf{X}.
\end{aligned}$$

The integrals are approximated using the Bayesian bootstrap procedure described in Section 3 of the manuscript. After fitting the linear model, $s = 1, ..., S$ samples are obtained from the joint posterior distribution of the model parameters, $\pi(\boldsymbol{\theta} \mid \mathcal{D}_{n_t})$. Let $\boldsymbol{\theta}_s$ represent the sth draw from this joint posterior distribution. For every row $i = 1, ..., n_t$ in the sample data, a value of $A_i = 1$ is assigned. Then for each $\boldsymbol{\theta}_s$ the following procedure is performed. The n_t values of the linear predictor $\mu(\boldsymbol{\theta}_s; A_i = 1, \mathbf{X}_i = \mathbf{x}_i)$ are calculated. A vector $\mathbf{w}_s = (w_{1,s}, ..., w_{n_t,s})$ is drawn from a Dirichlet($\mathbf{1}_{n_t}$) distribution. Using $\mathbf{w}_s$, the n_t values are then averaged, $\sum_{i=1}^{n_t} w_{i,s}\mu(\boldsymbol{\theta}_s; A_i = 1, \mathbf{X}_i = \mathbf{x}_i)$, which marginalizes them with respect to the observed $\mathbf{X} = \mathbf{x}$, yielding a single sample $\mu(\boldsymbol{\theta}_s; A = 1)$. This occurs for all $\boldsymbol{\theta}_s$ to yield S samples from the posterior distribution of $\mu(\boldsymbol{\theta}; A = 1)$. This entire process is then repeated for $A_i = 0$, to yield S samples from the posterior distribution of $\mu(\boldsymbol{\theta}; A = 0)$. These posterior samples are then subtracted to yield samples from the posterior distribution of $\gamma(\boldsymbol{\theta})$. A brief summary outline is included below.

1. Fit the linear regression model with identity link.
2. Obtain $s = 1, ..., S$ samples from the joint posterior distribution of the model parameters, $\pi(\boldsymbol{\theta} \mid \mathcal{D}_{n_t})$.
3. Create one copy of the sample data where $A_i = 1$ for all $i = 1, ..., n_t$.
4. For each $\boldsymbol{\theta}_s$, perform the following:
   (a) For each $i = 1, ..., n_t$, calculate $\mu(\boldsymbol{\theta}_s; A_i = 1, \mathbf{X}_i = \mathbf{x}_i)$.
   (b) Sample $\mathbf{w}_s = (w_{1,s}, ..., w_{n_t,s})$ from a Dirichlet($\mathbf{1}_{n_t}$) distribution.
   (c) Average these n_t values to marginalize with respect to the observed $\mathbf{X} = \mathbf{x}$:
   $\mu(\boldsymbol{\theta}_s; A_i = 1) = \sum_{i=1}^{n_t} w_{i,s}\mu(\boldsymbol{\theta}_s; A_i = 1, \mathbf{X}_i = \mathbf{X}_i)$.
5. The $s = 1, ..., S$ values of $\mu(\boldsymbol{\theta}_s; A = 1)$ are samples from the posterior of $\mu(\boldsymbol{\theta}; A = 1)$.
6. Repeat steps 3-4 for $A_i = 0$ to yield S samples from the posterior of $\mu(\boldsymbol{\theta}; A = 0)$.
7. Subtract to obtain S samples from the posterior of $\gamma(\boldsymbol{\theta})$.

A.2 Binary Outcome: Relative Risk

Letting Y be distributed as a Bernoulli random variable, where $Y = 1$ indicates an event occurs and $Y = 0$ indicates no event occurs, a marginal estimand of interest is the relative risk:

$$\gamma(\boldsymbol{\theta}) := \mu(\boldsymbol{\theta}; A = 1)/\mu(\boldsymbol{\theta}; A = 0)$$

where $\mu(\boldsymbol{\theta}; A = a) = E[Y \mid A = a; \boldsymbol{\theta}]$. Estimation proceeds assuming independent outcomes and the following model:

$$\begin{aligned} p(Y_i \mid A_i, \mathbf{X}_i, \boldsymbol{\theta}) &= \text{Bernoulli}(\mu(\boldsymbol{\theta}; A_i, \mathbf{X}_i)) \\ \mu(\boldsymbol{\theta}; A_i, \mathbf{X}_i) &= \text{logit}^{-1}(\beta_0 + \phi A_i + \mathbf{X}_i\boldsymbol{\beta} + (A_i \cdot \mathbf{Z}_i)\boldsymbol{\omega}) \\ \boldsymbol{\theta} &= \{\beta_0, \phi, \boldsymbol{\beta}, \boldsymbol{\omega}\} \end{aligned} \qquad \begin{aligned} \mathcal{L} &= \prod_{i=1}^{n_t} p(Y_i \mid A_i, \mathbf{X}_i, \boldsymbol{\theta}) \\ \pi(\boldsymbol{\theta} \mid \mathcal{D}_{n_t}) &\propto \prod_{i=1}^{n_t} p(Y_i \mid A_i, \mathbf{X}_i, \boldsymbol{\theta})p(\boldsymbol{\theta}). \end{aligned}$$

To obtain posterior samples of the marginal estimand from adjusted analyses, $s = 1, ..., S$ posterior samples from the inverse logit link function applied to the linear predictors under treatment and no treatment are marginalized and then divided:

$$\begin{aligned} \gamma(\boldsymbol{\theta}_s) &= \frac{\mu(\boldsymbol{\theta}_s; A = 1)}{\mu(\boldsymbol{\theta}_s; A = 0)} \\ &= \frac{\int_{\mathbf{X}} \mu(\boldsymbol{\theta}_s; A = 1, \mathbf{X})p(\mathbf{X})d(\mathbf{X})}{\int_{\mathbf{X}} \mu(\boldsymbol{\theta}_s; A = 0, \mathbf{X})p(\mathbf{X})d(\mathbf{X})} \\ &= \frac{\int_{\mathbf{X}} \text{logit}^{-1}\{\beta_{0,s} + \phi_s + \mathbf{X}\boldsymbol{\beta}_s + \mathbf{Z}\boldsymbol{\omega}_s\}p(\mathbf{X})d(\mathbf{X})}{\int_{\mathbf{X}} \text{logit}^{-1}\{\beta_{0,s} + \mathbf{X}\boldsymbol{\beta}_s\}p(\mathbf{X})d(\mathbf{X})} \end{aligned}$$

The integrals are approximated using the Bayesian bootstrap procedure described in Section 3 of the manuscript. After fitting the generalized linear model with logit link function corresponding to η, $s = 1, ..., S$ samples are obtained from the joint posterior distribution of the model parameters, $\pi(\boldsymbol{\theta} \mid \mathcal{D}_{n_t})$. Let $\boldsymbol{\theta}_s$ represent the sth draw from this joint posterior distribution. For every row $i = 1, ..., n_t$ in the sample data, a value of $A_i = 1$ is assigned. Then for each $\boldsymbol{\theta}_s$ the following procedure is performed. The n_t values of the indexed linear predictors are calculated and transformed by the inverse logit to yield samples from $\mu(\boldsymbol{\theta}_s; A_i = 1, \mathbf{X}_i = \mathbf{x}_i) = \text{logit}^{-1}(\beta_{0,s} + \phi_s A_i + \mathbf{X}_i\boldsymbol{\beta}_s + (A_i \cdot \mathbf{Z}_i)\boldsymbol{\omega}_s)$. A vector $\mathbf{w}_s = (w_{1,s}, ..., w_{n_t,s})$ is drawn from a Dirichlet($\mathbf{1}_{n_t}$) distribution. Using $\mathbf{w}_s$, the n_t values are then averaged, $\sum_{i=1}^{n_t} w_{i,s}\mu(\boldsymbol{\theta}_s; A_i = 1, \mathbf{X}_i = \mathbf{x}_i)$, which marginalizes them with respect to the observed $\mathbf{X} = \mathbf{x}$, yielding a single sample $\mu(\boldsymbol{\theta}_s; A = 1)$ from the posterior distribution of $\mu(\boldsymbol{\theta}; A = 1)$. This occurs for all $\boldsymbol{\theta}_s$ to yield S samples from the posterior distribution of $\mu(\boldsymbol{\theta}; A = 1)$. This entire process is then repeated for $A_i = 0$, to yield S samples from the posterior distribution of $\mu(\boldsymbol{\theta}; A = 0)$. These are then divided to yield S samples from the posterior distribution of $\gamma(\boldsymbol{\theta})$. A brief summary outline is included below.

1. Fit the logistic regression model.
2. Obtain $s = 1, ..., S$ samples from the joint posterior distribution of the model parameters, $\pi(\boldsymbol{\theta} \mid \mathcal{D}_{n_t})$.
3. Create one copy of the sample data where $A_i = 1$ for all $i = 1, ..., n_t$.
4. For each $\boldsymbol{\theta}_s$, perform the following:
   (a) For each $i = 1, ..., n_t$, calculate $\mu(\boldsymbol{\theta}_s; A_i = 1, \mathbf{X}_i = \mathbf{x}_i) = \text{logit}^{-1}(\beta_{0,s} + \phi_s A_i + \mathbf{X}_i\boldsymbol{\beta}_s + (A_i \cdot \mathbf{Z}_i)\boldsymbol{\omega}_s)$.
   (b) Sample $\mathbf{w}_s = (w_{1,s}, ..., w_{n_t,s})$ from a Dirichlet($\mathbf{1}_{n_t}$) distribution.
   (c) Average these n_t values to marginalize with respect to the observed $\mathbf{X} = \mathbf{x}$:
   $\mu(\boldsymbol{\theta}_s; A = 1) = \sum_{i=1}^{n_t} w_{i,s}\mu(\boldsymbol{\theta}_s; A_i = 1, \mathbf{X}_i = \mathbf{x}_i)$.
5. The $s = 1, ..., S$ values of $\mu(\boldsymbol{\theta}_s; A = 1)$ are samples from the posterior distribution of $\mu(\boldsymbol{\theta}; A = 1)$.
6. Repeat steps 3-4 for $A_i = 0$ to yield S samples from the posterior distribution of $\mu(\boldsymbol{\theta}; A = 0)$.
7. Divide $\mu(\boldsymbol{\theta}_s; A = 1)/\mu(\boldsymbol{\theta}_s; A = 0)$ for each s to obtain S samples from the posterior distribution of $\gamma(\boldsymbol{\theta})$.

A.3 Time-to-event Outcome: Marginalization of Conditional Hazard Ratio Estimates

Let $Y = \{T, \delta\}$ be defined as in the section for hazard ratios in the manuscript, where the target of inference is the marginal hazard ratio:

$$\begin{aligned} \gamma(\boldsymbol{\theta}) &= h(t \mid A = 1)/h(t \mid A = 0) \\ &= \log\{\mu(\boldsymbol{\theta}; A = 1)\}/\log\{\mu(\boldsymbol{\theta}; A = 0)\} \end{aligned}$$

where $\mu(\boldsymbol{\theta}; A = a) = S(t \mid A = a; \boldsymbol{\theta})$. Estimation proceeds assuming independent outcomes, no competing risks, and the following model:

$$\begin{aligned} h_i(t \mid A_i, \mathbf{X}_i) &= h_0(t)\exp(\eta_i) \\ \eta_i &= \phi A_i + \mathbf{X}_i\boldsymbol{\beta} + (A_i \cdot \mathbf{Z_i})\boldsymbol{\omega} \\ S_i(t \mid A_i, \mathbf{X}_i) &= \exp\left(-I(t; \boldsymbol{\psi}, \boldsymbol{k}, \delta)\exp(\eta_i)\right) \\ \boldsymbol{\theta} &= \{\boldsymbol{\psi}, \phi, \boldsymbol{\beta}, \boldsymbol{\omega}\} \end{aligned}$$

$$\begin{aligned} p(Y_i \mid A_i, \mathbf{X}_i, \boldsymbol{\theta}) &= S_i(T_i \mid A_i, \mathbf{X}_i)^{1-\delta_i} h_i(T_i \mid A_i, \mathbf{X}_i)^{\delta_i} \\ \mathcal{L} &= \prod_{i=1}^{n_t} p(Y_i \mid A_i, \mathbf{X}_i, \boldsymbol{\theta}) \\ \pi(\boldsymbol{\theta} \mid \mathcal{D}_{n_t}) &\propto \prod_{i=1}^{n_t} p(Y_i \mid A_i, \mathbf{X}_i, \boldsymbol{\theta}) p(\boldsymbol{\theta}). \end{aligned}$$

As the hazard ratio is non-collapsible, $s = 1, ..., S$ marginal posterior samples from adjusted analyses are obtained through marginalization of the log transformed survival probabilities:

$$\begin{aligned} \gamma(\boldsymbol{\theta}_s) &= \frac{h_s(t \mid A = 1)}{h_s(t \mid A = 0)} \\ &= \frac{\log\{\mu(\boldsymbol{\theta}_s; A = 1)\}}{\log\{\mu(\boldsymbol{\theta}_s; A = 0)\}} \\ &= \frac{\log\{\int_{\mathbf{X}} \mu(\boldsymbol{\theta}_s; A = 1, \mathbf{X}) p(\mathbf{X}) d\mathbf{X}\}}{\log\{\int_{\mathbf{X}} \mu(\boldsymbol{\theta}_s; A = 0, \mathbf{X}) p(\mathbf{X}) d\mathbf{X}\}} \\ &= \frac{\log\{\int_{\mathbf{X}} \exp\left[-I(t; \boldsymbol{\psi}_s, \boldsymbol{k}, \delta)\exp(\phi_s + \mathbf{X}\boldsymbol{\beta}_s + \mathbf{Z}\boldsymbol{\omega}_s)\right] p(\mathbf{X}) d\mathbf{X}\}}{\log\{\int_{\mathbf{X}} \exp\left[-I(t; \boldsymbol{\psi}_s, \boldsymbol{k}, \delta)\exp(\mathbf{X}\boldsymbol{\beta}_s)\right] p(\mathbf{X}) d\mathbf{X}\}}. \end{aligned}$$

Dividing log-transformed survival probabilities can be numerically unstable and is undefined for all t such that $S(t|A = a) \in \{0, 1\}$. Thus we use a more numerically stable identity [Stitelman et al., 2011, Remiro-Azócar et al., 2020]:

$$\begin{aligned} \log(\gamma(\boldsymbol{\theta}_s)) = \log\left(\frac{h_s(t|A = 1)}{h_s(t|A = 0)}\right) &= \log\left(\frac{-\log[\mu(\boldsymbol{\theta}_s; A = 1)]}{-\log[\mu(\boldsymbol{\theta}_s; A = 0)]}\right) \\ &= \log\{-\log[\mu(\boldsymbol{\theta}_s; A = 1)]\} - \log\{-\log[\mu(\boldsymbol{\theta}_s; A = 0)]\}. \end{aligned}$$

The integrals are approximated using the Bayesian bootstrap procedure described in Section 3 of the manuscript. After fitting the flexible, semi-parametric proportional hazards model, $s = 1, ..., S$ samples are obtained from the joint posterior distribution of the model parameters, $\pi(\boldsymbol{\theta} \mid \mathcal{D}_{n_t})$. Let $\boldsymbol{\theta}_s$ represent the sth draw from this joint posterior distribution. For every row $i = 1, ..., n_t$ in the sample data, a value of $A_i = 1$ is assigned. Then for each $\boldsymbol{\theta}_s$ the following procedure is performed. For the t corresponding to the time from the start of the trial to the current analysis, the n_t values of the indexed conditional survival probabilities, $\mu(\boldsymbol{\theta}_s; A_i = 1, \mathbf{X}_i = \mathbf{x}_i)$ are calculated. A vector $\mathbf{w}_s = (w_{1,s}, ..., w_{n_t,s})$ is drawn from a Dirichlet$(\mathbf{1}_{n_t})$ distribution. Using $\mathbf{w}_s$, the n_t values are then averaged, $\sum_{i=1}^{n_t} w_{i,s}\mu(\boldsymbol{\theta}_s; A_i = 1, \mathbf{X}_i = \mathbf{x}_i)$, which marginalizes them with respect to the observed $\mathbf{X} = \mathbf{x}$, yielding a single sample $\mu(\boldsymbol{\theta}_s; A = 1)$ from the posterior distribution of $\mu(\boldsymbol{\theta}; A = 1)$. For numerical stability, a $\log\{-\log[\cdot]\}$ transformation is applied to yield a single sample from the posterior distribution of $\log\{h(t \mid A = 1)\}$. This occurs for all $\boldsymbol{\theta}_s$ to yield S draws from the posterior distribution of $\log\{h(t \mid A = 1)\}$. This entire process is then repeated for $A_i = 0$, to yield S draws from the posterior distribution of $\log\{h(t \mid A = 0)\}$. These posterior draws are subtracted and then exponentiated to yield samples from the posterior distribution of the marginal hazard ratio $\gamma(\boldsymbol{\theta})$. A brief summary is below.

1. Fit a flexible semi-parametric proportional hazards model.
2. Obtain $s = 1, ..., S$ samples from the joint posterior distribution of the model parameters, $\pi(\boldsymbol{\theta} \mid \mathcal{D}_{n_t})$.
3. Create one copy of the sample data where $A_i = 1$ for all $i = 1, ..., n_t$.
4. For each $\boldsymbol{\theta}_s$, perform the following:
    (a) For each $i = 1, ..., n_t$, calculate the conditional survival probabilities at time t corresponding to the time from the start of the trial to the current analysis, $\mu(\boldsymbol{\theta}_s; A_i = 1, \mathbf{X}_i = \mathbf{x}_i)$.
    (b) Sample $\mathbf{w}_s = (w_{1,s}, ..., w_{n_t,s})$ from a Dirichlet$(\mathbf{1}_{n_t})$ distribution.
    (c) Average these n_t values to marginalize with respect to the observed $\mathbf{X} = \mathbf{x}$: $\mu(\boldsymbol{\theta}_s; A = 1) = \sum_{i=1}^{n_t} w_i\mu(\boldsymbol{\theta}_s; A_i = 1, \mathbf{X}_i = \mathbf{x}_i)$.
    (d) Apply a $\log\{-\log[\cdot]\}$ transformation to yield a single sample from the posterior distribution of $\log\{h(t \mid A = 1)\}$.

5. This yields S samples from the posterior distribution of $\log\{h(t \mid A = 1)\}$.
6. Repeat steps 3-4 for $A_i = 0$ to yield S samples from the posterior distribution of $\log\{h(t \mid A = 0)\}$.
7. Subtract and then exponentiate to obtain S samples from the posterior distribution of the marginal hazard ratio, $\gamma(\boldsymbol{\theta})$.

B Ascertainment of Marginal Estimand Values

B.1 Ascertainment of Marginal Relative Risk (Binary Outcome)

We first select a value of β_0 on the log-odds scale in the adjusted data generating models, such that the simulated datasets have a specific marginal control event risk (p_{ctr}). We then use β_0 and the obtained values of ϕ (i.e., those where the unadjusted model achieves 50% and 80% power, also on the log-odds scale) to select the reported value of the marginal relative risk.

To find β_0, let Y be a binary outcome. As a reminder, we define A as the treatment assignment indicator, where $A = 1$ means being assigned to the treatment group and $A = 0$ means being assigned to the control group. Let l be the number of participants assigned to control, k be the number of participants assigned to treatment, and $l + k = n$ be the total number of participants potentially enrolled in the trial. Let $\mathbf{X}_{n\times p}$ be the set of covariates used in the adjusted data generating model, and $\mathbf{X}_i$ be the row vector corresponding to the values of the covariates for the ith participant. Recall the marginal control event risk, p_{ctr}, is the risk of having an event in those assigned to control. Then p_{ctr} can be defined with respect to an adjusted data generating model as follows:

$$\begin{aligned}
p_{ctr} &= E[Y|A=0] \\
&\approx \frac{1}{l}\sum_{i=1}^{l} \hat{E}[Y_i|A_i=0,\mathbf{X}_i] \\
&= \frac{1}{l}\sum_{i=1}^{l} \text{logit}^{-1}\{\beta_0 + \phi(A_i=0) + \mathbf{X}_i\boldsymbol{\beta}\} \\
&= \frac{1}{l}\sum_{i=1}^{l} \text{logit}^{-1}\{\beta_0 + \mathbf{X}_i\boldsymbol{\beta}\} \\
0 &= \left[\sum_{i=1}^{l} \text{logit}^{-1}\{\beta_0 + \mathbf{X}_i\boldsymbol{\beta}\}\right] - l \times p_{ctr}
\end{aligned}$$

Given a fixed value of p_{ctr}, conditional covariate effects $\boldsymbol{\beta}$ on the log-odds scale, and initial simulation of the treatment assignment and covariate distributions, $\{A, \mathbf{X}\}$, β_0 can be optimized using the last line above (i.e., using uniroot() in R).

In the simulations for each relative risk value within each maximum sample size, 5,000 datasets (each with 5,000 participants) were generated using $\{\boldsymbol{\beta}, A, \mathbf{X}\}$ as described under the binary outcome data generating mechanism. From these, 5,000 values for β_0 were found and the mean of this distribution was selected as the value of β_0. Using this and the value of ϕ, 5,000 values for the marginal relative risk were obtained by dividing the proportion of events in those assigned to treatment ($\hat{E}[Y|A=1]$) by the proportion of events in those assigned to control ($\hat{E}[Y|A=0]$). The mean of this distribution was then reported as the value of the marginal relative risk corresponding to β_0 and ϕ.

B.2 Ascertainment of Marginal Hazard Ratio (Time-to-event Outcome)

Our goal is specify a value of the reported marginal hazard ratio which corresponds to the value of ϕ (on the log-hazard scale) used in the adjusted data generating models. Recall our assumption of proportional hazards, where the marginal hazard ratio is not time-dependent. Let $Y = \{T, \delta\}$ be defined as in the section for hazard ratios in the manuscript. Define A as the treatment assignment indicator, where $A = 1$ means being assigned to the treatment group and $A = 0$ means being assigned to the control group. Let t be the maximum duration of the trial and $P(T > t|A = 1) = S(t|A = 1)$ and $P(T > t|A = 0) = S(t|A = 0)$ be the survival probabilities at time t for those assigned to treatment and control, respectively. In the simulations for each hazard ratio value within each maximum sample size, 5,000 datasets (each with 5,000 participants) were generated using $\{\boldsymbol{\beta}, A, \mathbf{X}, t = 50\}$ as described under the time-to-event outcome data generating mechanism. For each dataset, the value of the marginal hazard ratio was calculated as:

$$\gamma = \exp\{\log(-\log[\hat{P}(T > 50|A = 1)]) - \log(-\log[\hat{P}(T > 50|A = 0)])\}$$

The mean of this distribution was then reported as the value of the marginal hazard ratio corresponding to ϕ.

C CCEDRN COVID-19 RCT Truncated Covariate Distributions

The following algorithm yields datasets from truncated Normal distribution F with correct post-truncation minimum $\wedge$, maximum $\vee$, $q1$, and $q3$ values and approximately correct post-truncation μ and σ values.

1. Determine maximum sample size of trial, max_ss.
2. Obtain reported summary statistics $\{q1, q3, \mu, \sigma\}$ and range $[\wedge, \vee]$.
3. Set plausible value of median $q2$ if not reported.
4. Set $n = 250 \times max\_ss$.
5. Select starting values for $\{\xi, \tau^2\}$.
6. Until $\mu^* \approx \mu$ and $\sigma^* \approx \sigma$:
   (a) Generate $i = 1, ..., n$ values of X_i from $N(\xi, \tau^2)$.
   (b) Discard $X_i \notin [min, max]$.
   (c) Sample $\frac{n}{4}$ values of $X_i \in [\wedge, q1]$.
   (d) Sample $\frac{n}{4}$ values of $X_i \in [q1, q2]$.
   (e) Sample $\frac{n}{4}$ values of $X_i \in [q2, q3]$.
   (f) Sample $\frac{n}{4}$ values of $X_i \in [q3, \vee]$.
   (g) Collect all and set μ^* and σ^* as the mean and standard deviation of the sampled values.
   (h) Update $\{\xi, \tau^2\}$ or break if $\mu^* \approx \mu$ and $\sigma^* \approx \sigma$.
7. To generate one dataset from F, use final values of $\{\xi, \tau^2\}$ to repeat process above, but sample max_ss values from the n collected values.

For age, the following summary statistics and simulation parameter values were used:

$$\{\wedge = 18, q1 = 39, q2 = 55, q3 = 70, \vee = 90, \mu = 54.7, \sigma = 19.8, \xi = 62, \tau = 40\}.$$

For respiratory rate, the following summary statistics and simulation parameter values were used:

$$\{\wedge = 12, q1 = 18, q2 = 20, q3 = 22, \vee = 40, \mu = 21.0, \sigma = 6.2, \xi = 30, \tau = 6\}.$$

D Summary graphics for bias and RMSE

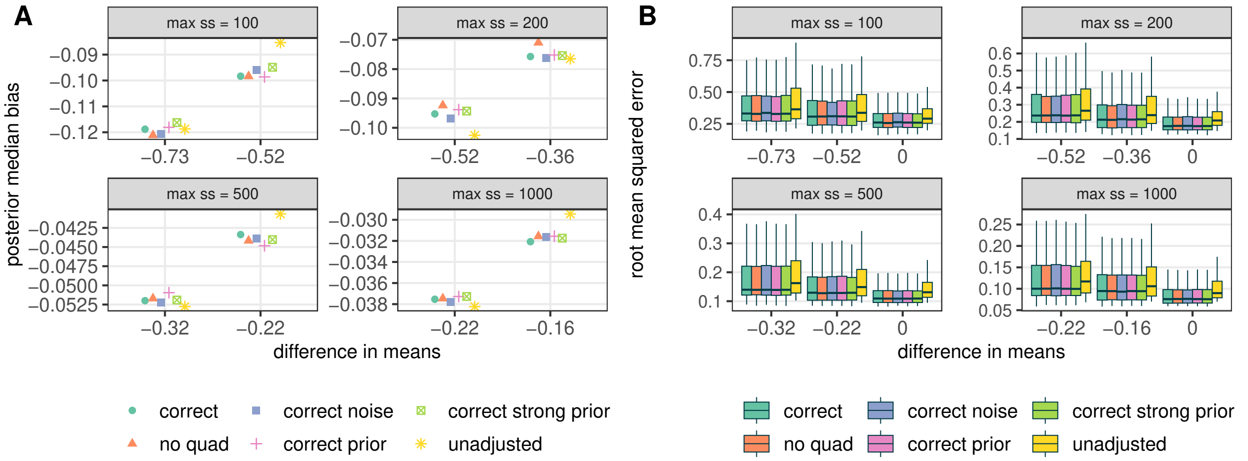

Figure D.1: Continuous outcome. A) Posterior median bias and B) root mean squared error. Panels correspond to various maximum sample sizes (max ss). Points are jittered horizontally.

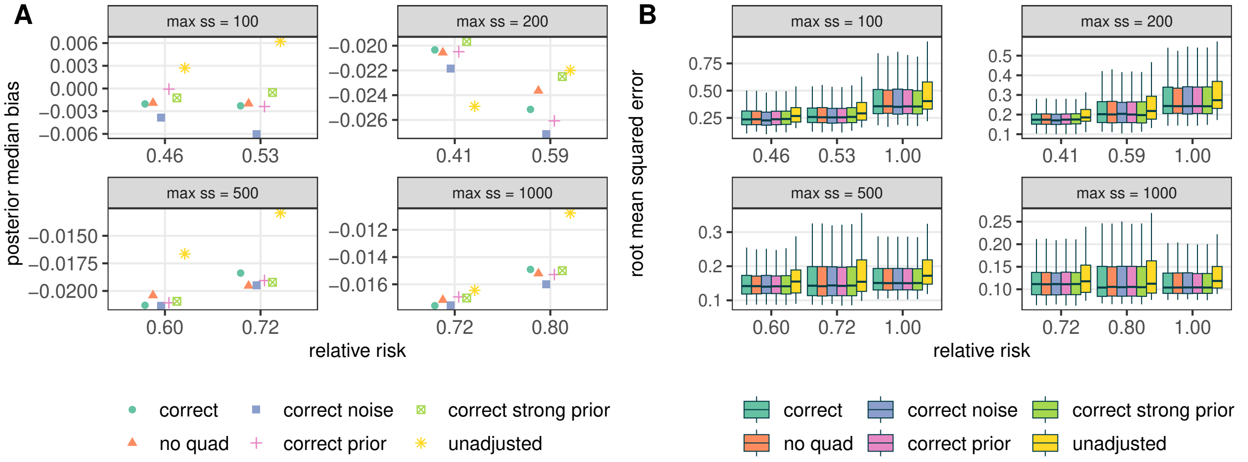

Figure D.2: Binary outcome. A) Posterior median bias and B) root mean squared error. Panels correspond to various maximum sample sizes (max ss). Points are jittered horizontally.

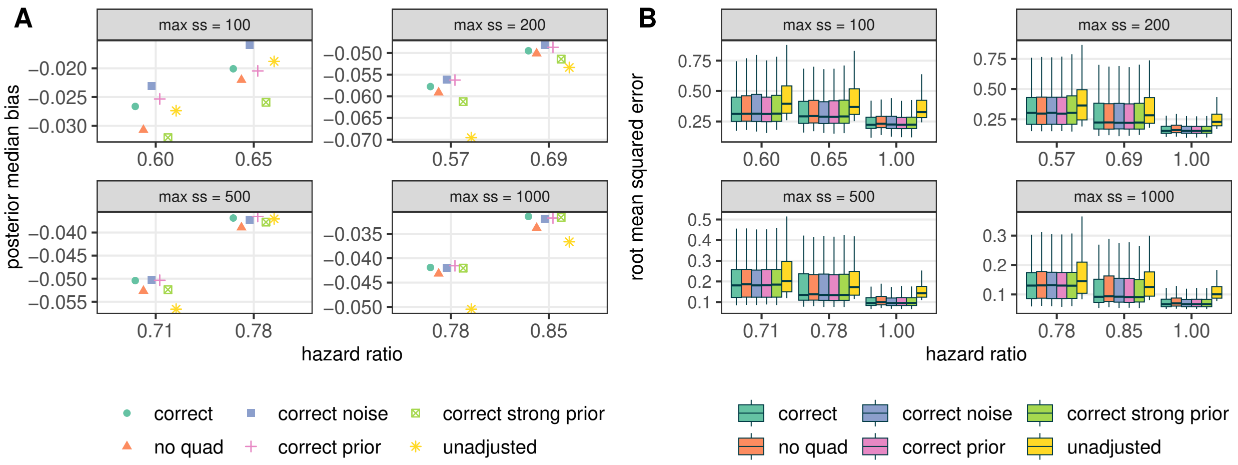

Figure D.3: Time-to-event outcome. A) Posterior median bias and B) root mean squared error. Panels correspond to various maximum sample sizes (max ss). Points are jittered horizontally.

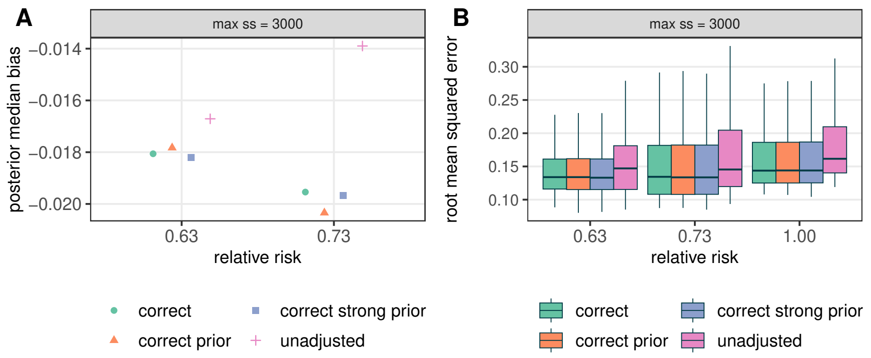

Figure D.4: CCEDRN-ADAPT COVID-19 trial with binary outcome. A) Posterior median bias and B) root mean squared error. Panels correspond to a maximum sample size (max ss) of 3,000. Points are jittered horizontally.

E Simulation for Informative Prior on Treatment Effect

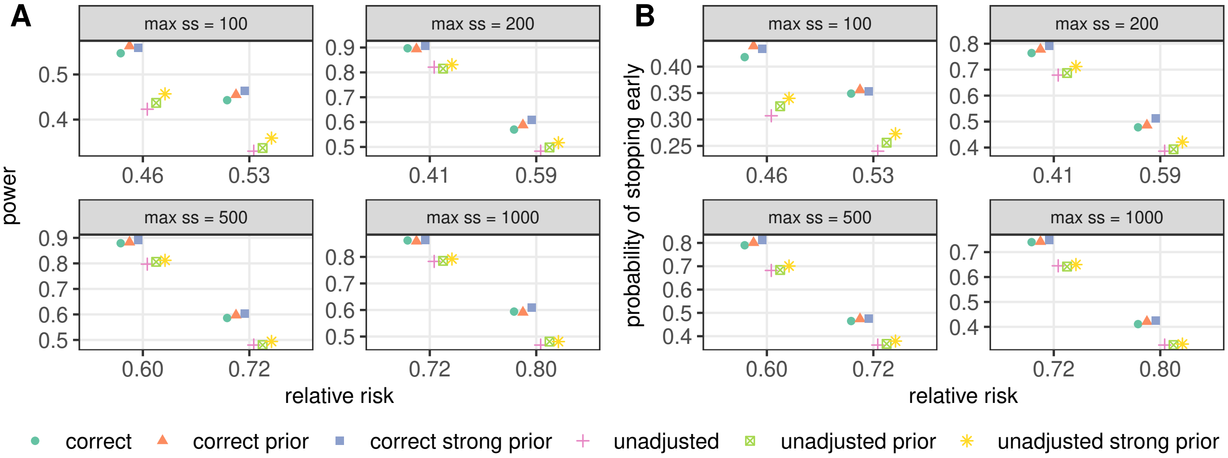

Figure E.1: Binary outcome. A) Power and B) probability of stopping early. Panels correspond to various maximum sample sizes (max ss). Points are jittered horizontally.

In this section, we consider the impact of incorporating informative prior information on the treatment effect for trials with binary endpoints. Six adjustment models are considered:

1. correct: $\beta_0 + \phi A + \beta_1 X_1 + \beta_2 X_2 + \beta_3 X_3 + \beta_4 X_3^2 + \beta_5 X_5$
2. correct prior: $\beta_0 + \phi A + \beta_1^\dagger X_1 + \beta_2^\dagger X_2 + \beta_3^\dagger X_3 + \beta_4^\dagger X_3^2 + \beta_5^\dagger X_5$
3. correct strong prior: $\beta_0 + \phi A + \beta_1^{\dagger\dagger} X_1 + \beta_2^{\dagger\dagger} X_2 + \beta_3^{\dagger\dagger} X_3 + \beta_4^{\dagger\dagger} X_3^2 + \beta_5^{\dagger\dagger} X_5$
4. unadjusted: $\beta_0 + \phi A$
5. unadjusted prior: $\beta_0 + \phi^\dagger A$
6. unadjusted strong prior: $\beta_0 + \phi^{\dagger\dagger} A$

The regression coefficients $\{\phi, \boldsymbol{\beta}, \boldsymbol{\beta}^\dagger, \boldsymbol{\beta}^{\dagger\dagger}\}$ and the functional forms of the *correct*, *correct prior*, *correct strong prior*, and *unadjusted* models are defined as previously described for the binary trials in the simulation study section of the manuscript. The *unadjusted prior* model includes a prior on the treatment indicator coefficient centered at the value used in the data generating mechanism where the *unadjusted* model achieves approximately 80% power (50% power for max ss = 100). The *unadjusted strong prior* model both centers and re-scales this prior to be more informative. The following priors are then used for the treatment indicator coefficients in the *unadjusted prior* and *unadjusted strong prior* models:

$$\phi^\dagger \sim \text{Normal}(c, 2.5/s_a)$$
$$\phi^{\dagger\dagger} \sim \text{Normal}(c, 1/s_a)$$

where $c = \{-1.21, -1.36, -0.82, -0.54\}$ for max ss $= \{100, 200, 500, 1000\}$. All other components for the binary trials remain as described in the simulation study section of the manuscript.

Results for power and the probability of stopping early are displayed in Figure E.1. Including stronger priors on the treatment effect may increase the power and probability of stopping early as compared to weakly informative priors. This hold for both the *correct* and *unadjusted* model variants and is most beneficial for smaller sample sizes. However, this comes at the cost of inflated type 1 error (T1E), with the greatest inflation occurring for the smaller maximum sample sizes (Table E.6). Both the type 1 error inflation and increase in power become less pronounced in the trials with larger maximum sample sizes where the priors are dominated by the data.

Table E.6: Binary outcome. Type 1 error rate (T1E), bias under the null (Bias*), and expected sample size at three different values of the marginal relative risk (γ).

	Maximum sample size = 100					Maximum sample size = 200				
			Expected sample size					Expected sample size		
Adjustment model	T1E	Bias*	$\gamma = 1$	$\gamma = 0.53$	$\gamma = 0.46$	T1E	Bias*	$\gamma = 1$	$\gamma = 0.59$	$\gamma = 0.41$
correct	0.063	0.031	97.0	86.3	83.6	0.036	0.021	196.8	162.5	138.3
correct prior	0.071	0.021	96.5	85.7	82.2	0.038	0.017	196.5	161.9	136.5
correct strong prior	0.063	-0.014	97.3	86.6	83.8	0.046	-0.006	195.7	159.2	134.9
unadjusted	0.034	0.058	98.6	90.7	88.3	0.031	0.025	198.0	171.4	147.2
unadjusted prior	0.034	0.051	98.6	90.0	87.4	0.032	0.021	197.9	170.8	146.2
unadjusted strong prior	0.041	0.009	98.4	89.6	86.8	0.034	0.001	197.6	167.6	142.8
	Maximum sample size = 500					Maximum sample size = 1000				
			Expected sample size					Expected sample size		
Adjustment model	T1E	Bias*	$\gamma = 1$	$\gamma = 0.72$	$\gamma = 0.60$	T1E	Bias*	$\gamma = 1$	$\gamma = 0.80$	$\gamma = 0.72$
correct	0.028	0.010	493.7	404.9	334.9	0.022	0.008	992.0	823.2	681.5
correct prior	0.028	0.009	494.1	402.9	334.9	0.021	0.007	991.7	818.9	680.3
correct strong prior	0.028	0.004	493.7	401.2	329.9	0.023	0.005	990.5	813.9	675.7
unadjusted	0.026	0.016	494.6	426.0	367.0	0.024	0.010	990.2	859.6	734.2
unadjusted prior	0.029	0.015	493.4	426.7	364.9	0.024	0.009	989.5	858.6	733.7
unadjusted strong prior	0.031	0.009	492.6	423.0	362.7	0.023	0.007	989.5	858.1	733.9

F Bias From Overestimation in Trials which Stop Early for Superiority

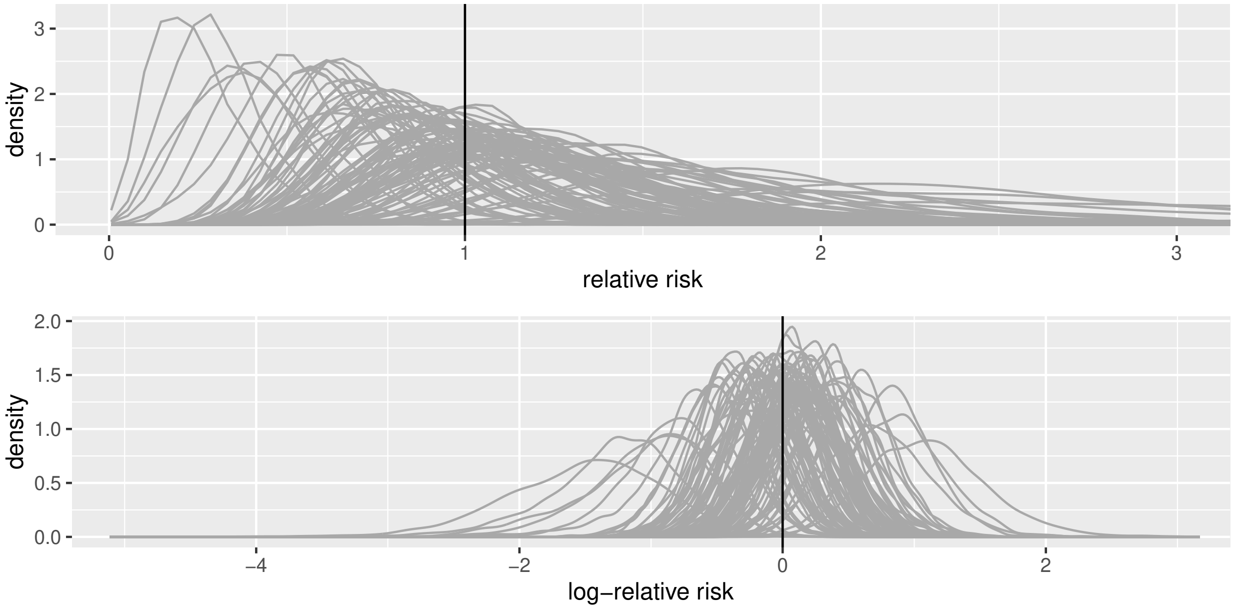

Figure F.1: 100 posterior distributions of the relative risk (top) and log-relative risk (bottom) for a trial with a binary endpoint and maximum sample size of 100. Vertical line represents the null treatment effect.

[Walter et al., 2019] shows that overestimation is to be expected when trials permit early stopping for superiority. They consider the case of frequentist group-sequential designs and compare three different stopping rules which differ in how the overall α is divided among the interim and final analyses. The Pocock, O'Brien, and Fleming (PCK) stopping rule evenly divides α across all analyses (interim and final) keeping the stringency of the stopping criteria constant. This is the frequentist group sequential stopping rule most like the Bayesian stopping rule employed in the current manuscript, where a single value for the upper probability threshold u is used (u=0.99), thereby also keeping the stringency of the stopping rule constant across all interim and final analyses. It is shown that overestimation is expected for the PCK stopping rule, and so we conclude it should also be expected for the Bayesian stopping rule employed here, thus inducing the observed bias in the treatment effect under the simulation scenarios. In Section 3.1 of [Walter et al., 2019], the authors discuss observing greater over-estimation for the Haybittle and Peto (HP) stopping rule as compared to the PCK stopping rule. They state "rules (such as HP) that have a more stringent threshold for stopping involve a greater risk of over-estimation if the rule is actually invoked." This suggests that Bayesian stopping rules which have more stringent initial stopping criteria may lead to increased bias as compared to the stopping rules employed in the current manuscript, though we do not explore this further here.

Considering bias under the null, the difference in signs between the continuous endpoint versus the binary and time-to-event endpoints reflects the lower bounds of the estimands. The difference in means under the continuous endpoint is unbounded below, whereas the relative risk and hazard ratios are bounded below by zero. When bias is calculated on the log-relative risk and log-hazard ratio scales, most values for bias under the null for both the binary and time-to-event endpoints become negative as well, with greater bias for smaller sample sizes as in the continuous endpoint. This is explained visually in Figure F.1 and F.2, where 100 posterior distributions (Figure F.1) and posterior medians (Figure F.2) have been plotted for the null treatment effect for a trial with a binary endpoint under a maximum sample size of 100. The vertical lines represent the null values used for calculation of bias.

In the top panel of Figure F.1 on the relative risk scale, we see many right-skewed posteriors which lead to some posterior median estimates which are much greater than the null (Figure F.2, top panel). This induces positive bias under the null. When we move to the log scale, the right-skewed distributions become more normal in shape and are more centered around the null, but some of the posteriors which were closer to the lower boundary of 0 on the relative risk scale become left-skewed (Figure F.1, bottom panel). This results in some posterior medians becoming much less than the null (Figure F.2, bottom panel) which leads to negative bias under the null as in the continuous endpoint case. When calculating bias for non-null treatment effects on the relative risk and hazard ratio scales, the posteriors are pushed further toward 0 than in the figures included in this section which is why they can still attain negative values and clearly exhibit overestimation in these cases.

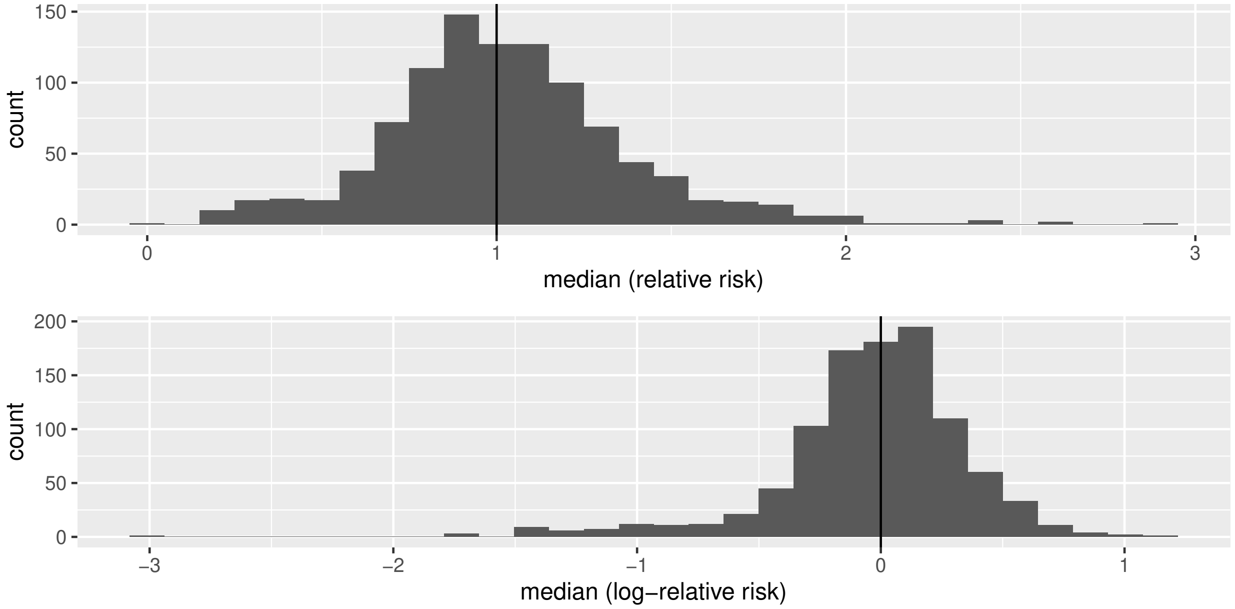

Figure F.2: 100 posterior medians of the relative risk (top) and log-relative risk (bottom) for a trial with a binary endpoint and maximum sample size of 100. Vertical line represents the null treatment effect.

G Example of the Non-Collapsibility of the Odds Ratio

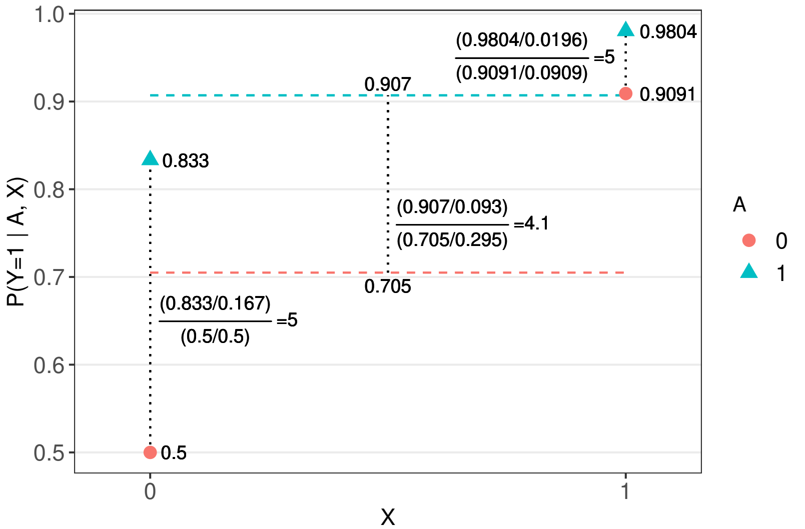

Figure G.1: Example of non-collapsibility of the odds ratio. Colored circles and triangles correspond to values of risk under treatment or control for different values of X.

We consider a slightly modified version of the example provided in [Daniel et al., 2021]. Consider a RCT with a binary endpoint following the logistic regression model below which contains a binary covariate X and binary treatment indicator A, and where $P(X = 1) = P(A = 1) = 0.5$. Define $\phi = \log(5)$, $\beta = \log(10)$, and $\boldsymbol{\theta} = \{\phi, \beta\}$. We assume the following model:

$$\text{logit}(P(Y = 1 \mid A, X)) = \phi A + \beta X.$$

In this example, the conditional odds ratio for those who are treated versus untreated is 5, regardless of the value of X. To see this, define $\mu(\boldsymbol{\theta}; A, X) = P(Y = 1 \mid A, X) = \text{logit}^{-1}(\phi A + \beta X)$. For $X = 0$, we have $\mu(\boldsymbol{\theta}; A = 1, X = 0) = 0.833$ and that $1 - \mu(\boldsymbol{\theta}; A = 1, X = 0) = 0.167$ yielding the odds for the event in those who are treated to be $\mu(\boldsymbol{\theta}; A = 1, X = 0)/1 - \mu(\boldsymbol{\theta}; A = 1, X = 0) = 0.833/0.167 = 5$. For those who are untreated, we have $\mu(\boldsymbol{\theta}; A = 0, X = 0) = 0.5$ and that $1 - \mu(\boldsymbol{\theta}; A = 0, X = 0) = 0.5$ yielding odds of $\mu(\boldsymbol{\theta}; A = 0, X = 0)/1 - \mu(\boldsymbol{\theta}; A = 0, X = 0) = 0.5/0.5 = 1$. Dividing these yields a conditional odds ratio for those who are treated versus untreated under $X = 0$ to be $5/1 = 5$. Similar calculations for $X = 1$ yield $\mu(\boldsymbol{\theta}; A = 1, X = 1) = 0.9804$, $1 - \mu(\boldsymbol{\theta}; A = 1, X = 1) = 0.0196$ yielding the odds for the event in those who are treated to be $0.9804/0.0196 = 50$. For those who are untreated, we have $\mu(\boldsymbol{\theta}; A = 0, X = 1) = 0.9091$ and that $1 - \mu(\boldsymbol{\theta}; A = 0, X = 1) = 0.0909$ yielding odds of $0.9091/0.0909 = 10$. Dividing these yields a conditional odds ratio for those who are treated versus untreated under $X = 1$ to be $50/10 = 5$. In Figure G.1, we that these conditional odds ratios correspond to a vertical comparison of the risks under the treatment assignments A for either value of X (dotted vertical lines). To obtain the marginal odds ratio, we must average these risks with respect to the distribution of X. This yields the horizontal dashed lines (colored by value of A) where $\mu(\boldsymbol{\theta}; A = 1) = 0.5(0.833) + 0.5(0.980) = 0.907$ and $\mu(\boldsymbol{\theta}; A = 0) = 0.5(0.5) + 0.5(0.909) = 0.705$. The marginal odds ratio then corresponds to a vertical comparison of these horizontal lines. Doing so yields a marginal odds ratio of $\gamma(\boldsymbol{\theta}) = (0.907/0.093)/(0.705/0.295) = 4.1$. We see that the marginal odds ratio is not equal to the conditional odds ratio, and thus the odds ratio is non-collapsible.

This same example can be viewed using two-by-two tables, where the cells contain the proportions expected under each combination of treatment assignment and covariate value.

X = 0

	$A = 1$	$A = 0$	$P(Y = y)$
$Y = 1$	0.8333333	0.5	0.667
$Y = 0$	0.16666667	0.5	0.333
$P(A = a)$	0.5	0.5	1

X = 1

	$A = 1$	$A = 0$	$P(Y = y)$
$Y = 1$	0.9803922	0.9090909	0.945
$Y = 0$	0.01960784	0.09090909	0.055
$P(A = a)$	0.5	0.5	1

The conditional odds ratio from each table is 5. Below, we consider the marginal table with proportions expected under each combination of treatment assignment and covariate value. This is found by averaging the risk values in the conditional tables with respect to the distribution of X, where we recall that $P(X = 1) = 0.5$.

	$A=1$	$A=0$	$P(Y=y)$
$Y=1$	0.90686275	0.70454545	0.806
$Y=0$	0.09313725	0.29545455	0.194
$P(A=a)$	0.5	0.5	1

The marginal odds ratio from the table above is 4.1. We observe that the true conditional and marginal odds ratios are not equal, thus showing the odds ratio is non-collapsible.
